# The Clinical Effectiveness of Blended Cognitive Behavioral Therapy Compared With Face-to-Face Cognitive Behavioral Therapy for Adult Depression: Randomized Controlled Noninferiority Trial

**DOI:** 10.2196/36577

**Published:** 2022-09-07

**Authors:** Kim Mathiasen, Tonny E Andersen, Mia Beck Lichtenstein, Lars Holger Ehlers, Heleen Riper, Annet Kleiboer, Kirsten K Roessler

**Affiliations:** 1 Research Unit for Telepsychiatry and E-Mental Health Department of Clinical Research University of Southern Denmark Odense Denmark; 2 Department of Psychology University of Southern Denmark Odense Denmark; 3 Danish Center for Healthcare Improvements Aalborg University Aalborg Denmark; 4 Nordic Institute of Health Economics Aarhus Denmark; 5 Department of Clinical, Neuro and Developmental Psychology Vrije Universiteit Amsterdam Amsterdam Netherlands

**Keywords:** depression, depressive disorder, major, cognitive therapy, CBT, treatment outcome, blended care, blended cognitive behavioral therapy, effectiveness, Denmark

## Abstract

**Background:**

Internet-based cognitive behavioral therapy (iCBT) has been demonstrated to be cost- and clinically effective. There is a need, however, for increased therapist contact for some patient groups. Combining iCBT with traditional face-to-face (FtF) consultations in a blended format may produce a new treatment format (B-CBT) with multiple benefits from both traditional CBT and iCBT, such as individual adaptation, lower costs than traditional therapy, wide geographical and temporal availability, and possibly lower threshold to implementation.

**Objective:**

The primary aim of this study is to compare directly the clinical effectiveness of B-CBT with FtF-CBT for adult major depressive disorder.

**Methods:**

A 2-arm randomized controlled noninferiority trial compared B-CBT for adult depression with treatment as usual (TAU). The trial was researcher blinded (unblinded for participants and clinicians). B-CBT comprised 6 sessions of FtF-CBT alternated with 6-8 web-based CBT self-help modules. TAU comprised 12 sessions of FtF-CBT. All participants were aged 18 or older and met the diagnostic criteria for major depressive disorder and were recruited via a national iCBT clinic. The primary outcome was change in depression severity on the 9-item Patient Health Questionnaire (PHQ-9). Secondary analyses included client satisfaction (8-item Client Satisfaction Questionnaire [CSQ-8]), patient expectancy (Credibility and Expectancy Questionnaire [CEQ]), and working (Working Alliance Inventory [WAI] and Technical Alliance Inventory [TAI]). The primary outcome was analyzed by a mixed effects model including all available data from baseline, weekly measures, 3-, 6, and 12-month follow-up.

**Results:**

A total of 76 individuals were randomized, with 38 allocated to each treatment group. Age ranged from 18 to 71 years (SD 13.96) with 56 (74%) females. Attrition rate was 20% (n=15), which was less in the FtF-CBT group (n=6, 16%) than in the B-CBT group (n=9, 24%). As many as 53 (70%) completed 9 or more sessions almost equally distributed between the groups (nFtF-CBT=27, 71%; nB-CBT=26, 68%). PHQ-9 reduced 11.38 points in the FtF-CBT group and 8.10 in the B-CBT group. At 6 months, the mean difference was a mere 0.17 points. The primary analyses confirmed large and significant within-group reductions in both groups (FtF-CBT: β=–.03; standard error [SE] 0.00; *P*<.001 and B-CBT: β=–.02; SE 0.00; *P*<.001). A small but significant interaction effect was observed between groups (β=.01; SE 0.00; *P*=.03). Employment status influenced the outcome differently between groups, where the B-CBT group was seen to profit more from not being full-time employed than the FtF group.

**Conclusions:**

With large within-group effects in both treatment arms, the study demonstrated feasibility of B-CBT in Denmark. At 6 months’ follow-up, there appeared to be no difference between the 2 treatment formats, with a small but nonsignificant difference at 12 months. The study seems to demonstrate that B-CBT is capable of producing treatment effects that are close to FtF-CBT and that completion rates and satisfaction rates were comparable between groups. However, the study was limited by small sample size and should be interpreted with caution.

**Trial Registration:**

ClinicalTrials.gov NCT02796573; https://clinicaltrials.gov/ct2/show/NCT02796573

**International Registered Report Identifier (IRRID):**

RR2-10.1186/s12888-016-1140-y

## Introduction

### Background

Depression is a prevalent and disabling disorder with a high risk of relapse and large individual and societal costs [1-6]. Effective treatments do exist [7], although a large gap is seen between the need for and use of treatments [8]. This gap has led researchers to explore alternative modes of treatment delivery. One such novel treatment format is internet-based cognitive behavioral therapy (iCBT) [9-17], in which the patient is administered access to an online treatment program based on CBT. The highest clinical effect is seen when clinical guidance is provided during the course of treatment [18-20]. However, despite the evidence for the effect of guided iCBT, there is a need for increased therapist contact among some patient groups as well as a need to provide a treatment format, which is more compatible with, and thus easier to implement in, the existing health care services [21-24].

Combining iCBT with traditional face-to-face (FtF) consultations in a blended CBT format (B-CBT), in which both online components and FtF sessions are included in 1 coherent CBT protocol, may alleviate some of the difficulties associated with iCBT for depression, while preserving some of the advantages of both iCBT and FtF-CBT alike. First, by including FtF sessions, the therapist can individualize the therapy taking the idiosyncratic case formulation of the patient, the specific disorder, and possible comorbidity into account. Second, as B-CBT in the format tested in this study only provides half the number of sessions as traditional FtF-CBT, the capacity of the treating clinician is increased compared with traditional CBT. Third, the burden and cost of travel by the patient can be reduced compared with FtF-CBT. Fourth, the online modules are available at the time and place needed by the patients—and they can be re-viewed multiple times. Fifth, the inherently structured format of the online modules ensures high treatment fidelity, for example, by delivering the same psychoeducation and exercises to all patients. Sixth, one of the principal barriers for the uptake of iCBT seems to be skepticism concerning allotting the majority of therapy to a computer [25], a barrier possibly alleviated by the B-CBT [26]. Finally, the blended format is more compatible with the existing health care services and as a consequence should be easier to implement than iCBT [27].

Few studies have investigated the use of blended care combining internet-based psychotherapeutic modules and FtF sessions into 1 coherent treatment manual to treat adult depression [28-31]. Generally, however, they do indicate positive outcomes. In a randomized controlled trial conducted in primary care in Tromsø, Norway, clinical psychologists delivered 30-minute sessions following each online module [29]. They were able to document a significant difference with a moderate to large effect size (*d*=0.65) on depressive symptoms (Beck Depression Inventory II [BDI-II]) favoring blended care over waiting list. The intervention predominantly received positive evaluations suggesting acceptability and satisfaction with the treatment. In addition, a qualitative study found that the FtF consultations increased motivation to persist with the iCBT program [32]. Another recent example is the development and initial evaluation of a program for B-CBT in The Netherlands. This was tested at an outpatient clinic of a specialized mental health care center in Amsterdam. The study was designed as a feasibility study and included only 9 patients. However, the patients perceived the intervention as positive, although the authors rightly noted that no conclusion can be derived from such a small sample [31]. A cohort study from the United States found a significant and large reduction in symptoms of depression but had no comparison group [33].

In this study we compared directly the clinical effect on adult depression of B-CBT and FtF-CBT in a randomized, controlled, noninferiority study in parallel groups, recruiting from a routine care iCBT clinic in the Region of Southern Denmark.

### Aims and Hypotheses

The primary aim of this study was to compare the clinical effectiveness of B-CBT for major depressive disorder in adults with treatment as usual (TAU) defined as 12 sessions of FtF-CBT. It is hypothesized that B-CBT will be no less clinically effective than FtF-CBT, and that it will be acceptable and satisfactory to patients and clinicians.

## Methods

### Design

The study was a randomized, controlled, noninferiority trial comparing B-CBT with FtF-CBT. It was part of the research program e-Mental Health Research (ENTER) located in and coordinated from the Centre for Telepsychiatry in the Mental Health Services of Southern Denmark, Odense. Additionally, this study was affiliated with the European Union (EU) study E-COMPARED [34]. However, the E-COMPARED study ended prior to this, and thus does not include the total sample. In this article we explore the full data set of the trial.

### Ethics Approval

The trial was approved by the Ethics Committee of the Region of Southern Denmark (registration number S-20150150) prior to instigation. The trial followed the Declaration of Helsinki on Ethical Principles for Medical Research Involving Human Subjects [35]. All participants received both written and oral information about the trial and signed written informed consent before entering the study.

### Trial Registration

The trial was registered with ClinicalTrials.gov NCT02796573. The trial protocol was published previously [36].

### Study Funding

Funding was granted from the Research Fund of the Mental Health Services of Southern Denmark, and from the Innovation Fund Denmark, as part of the project ENTER (ID: 5159-00002B). Both are public funds. None of the funds have had any role in the design of the study nor in the collection, analysis or interpretation of the data, or writing of the manuscript.

### Participants

#### Eligibility Criteria

All participants were 18 years of age or older and met the diagnostic criteria for major depressive disorder according to the Diagnostic and Statistical Manual of Mental Disorders 4th edition text revision (DSM-IV-TR) [37] as assessed by clinical psychologists. The diagnosis was confirmed by the research team using the semistructured interview Mini-International Neuropsychiatric Interview version 5.0 (M.I.N.I.) [38]. Furthermore, a score of at least five on the 9-item Patient Health Questionnaire-9 (PHQ-9) [39,40] was required. Patients were excluded in case of current high risk of suicide or if they had a comorbid substance dependence, bipolar disorder, psychotic illness, or obsessive-compulsive disorder. Additionally, participants were excluded if they concurrently received psychological treatment for depression. They were also required to comprehend the Danish language and have access to a personal computer and internet connection. Finally, they needed to be able and willing to travel to the physical location of the trial even if they were randomized to the FtF condition.

#### Recruitment

Participants were recruited from March 1, 2016, to April 1, 2018, from the iCBT clinic “Internetpsykiatrien,” which is situated within secondary mental health care (Centre for Telepsychiatry) at the Mental Health Services of the Region of Southern Denmark [41,42]. Internetpsykiatrien offers guided iCBT treatment for anxiety and depression with self-referral. Psychologists or master students in psychology interviewed all participants using M.I.N.I. [38] to confirm diagnosis. The interviews were administered either FtF or by telephone. In case the participants were on antidepressant medication, they were asked to keep it stable during treatment if possible. They were asked to report any changes in medication to the research team. Access to the program was provided by the research team.

### Randomization and Blinding

An independent researcher from the EU study E-COMPARED [34], who was not involved in the trial, performed the randomization at an individual level, stratified by country after eligibility and baseline measurement. A random number generator (Random Allocation Software) was applied with an allocation ratio of 1:1. Block randomization was used with block sizes varying from 8 to 14 allocations per block.

It was not possible to blind the patients nor the treating clinicians to the allocated treatment. However, those assessing the participants were blinded to allocation as were the researchers and statisticians involved up until the point of interpretation of the results. Some questionnaires were only administered to the B-CBT group and were kept in a separate data set.

### Interventions

#### Blended Treatment (B-CBT)

In the blended condition, 6 individual FtF-CBT sessions were alternated with 6-8 online CBT modules delivered through an internet-based treatment program. The FtF consultations were provided by a psychologist at the Centre for Telepsychiatry with physical presence by the participants and the therapists.

The program (NoDep) was previously developed (2015) as part of a public private innovation project between The Region of Southern Denmark and Context Consulting. It was based on CBT for depression and included 6 mandatory modules and 2 optional ones. The core components of the mandatory modules were psychoeducation, cognitive restructuring, behavioral activation, behavior experiments, and relapse prevention. The optional modules comprised coping with rumination and restructuring of core beliefs. All online modules were introduced in the FtF sessions. Modules the participants had previously worked with could be addressed in the FtF sessions if needed. The decision as to whether any optional modules need to be added was taken jointly by the patient and the psychologist based on patient needs, motivation, and possible time constraints. See Table 1 for an overview of the intervention. All modules were delivered via multimedia elements including video, audio, interactive exercises, calendar, and PDF summaries. The program had a build-in workflow predetermining the order in which the modules were presented. All data were stored in Europe and encrypted during storage and transmission.

**Table 1:** Overview of interventions.

Intervention and session number		Format of delivery	Content	Example of exercise
**B-CBT^a^**				
	1	FtF^b^	Introduction and psychoeducation about depression and the treatment	Find a helper
	2	Online module	Introduction to the program, psychoeducation about depression, and goals for the treatment	Problem/goal list
	3	FtF	Idiosyncratic model of the disorder	Cognitive case formulation
	4	Online module	Psychoeducation about behavior in depression	Activity registration
	5	FtF	Accordance between personal values and behavior. Introduction to cognitive restructuring	Simple exercise for cognitive restructuring
	6	Online module	Changing behavior based on activity registration and personal values	Activity planning
	7	FtF	Psychoeducation about negative automatic thoughts and cognitive restructuring	Cognitive restructuring exercise
	8	Online module	Psychoeducation about negative automatic thoughts and cognitive restructuring	Cognitive restructuring exercise
	9	FtF	Psychoeducation about behavioral experiments. Decision is made as to whether to include either or both of the extra modules	Behavioral experiment
	10	Online module (A, B)	Behavioral experiments (A: psychoeducation about core beliefs, B: coping with rumination)	Behavioral experiment (A: challenge core beliefs; B: test 3 techniques for coping with rumination)
	11	FtF	Summing up, relapse prevention	Continuation of preferred exercises
	12	Online module	Summing up, relapse prevention	Personal relapse prevention plan
**TAU^c^**				
	1	FtF	Introduction and psychoeducation about depression and the treatment	Find a helper
	2	FtF	Psychoeducation and goals for the treatment	Problem/goal list
	3	FtF	Idiosyncratic model of the disorder	Cognitive case formulation
	4	FtF	Psychoeducation about behavior in depression	Activity registration
	5	FtF	Accordance between personal values and behavior. Introduction to cognitive restructuring	Simple exercise for cognitive restructuring
	6	FtF	Changing behavior based on activity registration and personal values	Activity planning
	7	FtF	Psychoeducation about negative automatic thoughts and cognitive restructuring	Cognitive restructuring exercise
	8	FtF	Psychoeducation about negative automatic thoughts and cognitive restructuring	Cognitive restructuring exercise
	9	FtF	Psychoeducation about behavioral experiments	Behavioral experiment
	10	FtF	Psychoeducation about core beliefs or continue working on behavioral experiments	Challenge core beliefs or behavioral experiment
	11	FtF	Psychoeducation about rumination or beginning of relapse prevention	Test 3 techniques to cope with rumination or start personal relapse prevention plan and continuation of preferred exercise
	12	FtF	Summing up, relapse prevention	Personal relapse prevention plan

^a^B-CBT: blended cognitive behavioral therapy.

^b^FtF: face-to-face.

^c^TAU: treatment as usual.

To provide technical support to the participants, the existing procedures at the Centre for Telepsychiatry were used, which consisted of 2 levels: the first was handled by the clinicians, the second went through an error report system to the company that provided the software (Context Consulting).

No important changes were made to the program or the protocol during the trial.

#### Treatment as Usual

TAU defined as 12 sessions of FtF-CBT was also provided by a psychologist at the Centre for Telepsychiatry with physical presence and comprised the same core components as the B-CBT condition. Additionally, interventions on core beliefs and rumination could be included according to the same criteria as in the B-CBT condition. See Table 1 for an overview of the intervention.

Both treatment conditions were described in a single common treatment protocol, thus ensuring similar treatment content and order of interventions across the 2 groups. They were both intended to last approximately 12 weeks.

#### Safety Procedures

Patients in either condition were monitored weekly for symptoms of depression including suicidal ideation and intent. In case a participant’s condition deteriorated or showed signs of suicidal intent, a standard assessment procedure used in all of the secondary mental health care services in the Region of Southern Denmark was conducted. The patient was discontinued if necessary and referred to other relevant treatment.

#### Adherence and Fidelity

Licensed clinical psychologists or psychologists under supervision of the primary researcher (KM), who is also a licensed clinical psychologist, delivered all FtF consultations. To assess clinician fidelity [43], all FtF sessions were audio recorded and 20 sessions were randomly selected and evaluated by an external clinical expert (clinical psychologist and PhD with many years’ experience). Clinician adherence was defined as the number of *pre*scribed interventions that were *pro*scribed in the session. The level of agreement between the 2 were rated on a 5-point scale ranging from none (1) to all (5) [44].

To increase adherence, participants received automated reminders of homework assignments and questionnaires. Furthermore, in case a participant was inactive, he or she would be contacted by telephone or email. Additionally, in case a participant was unwilling or unable to engage with the program at home, a computer was set up at the clinic, for participants to engage with the online program on-site. This was never used, however.

### Outcome Measurements

After consent was granted, baseline measures were administered prior to randomization. Follow-up measurements were conducted 3, 6, and 12 months after baseline. Additionally, weekly measures were provided during treatment. The questionnaire packages were administered online using a secure web application for building and managing online surveys (REDCap), except for the weekly monitoring of the B-CBT group, for which the packages were administered automatically by the treatment program.

Data were stored by the Odense Patient data Exploratory Network (OPEN) [45]. Data were collected, transferred, and stored securely electronically as approved by the Danish Data Protection Agency (journal number: 14/26634, registration number: 2008-58-0035).

The PHQ-9 [39] was used as the primary outcome measure. The PHQ-9 is a 9-item questionnaire developed to measure depressive symptomatology in the primary health care sector. The 9 items are each scored on a 0-3-point scale with the total score ranging from 0 to 27, with higher scores indicating more severe depression. The authors suggest using cut-off points of 5, 10, and 15 for mild, moderate, and severe levels of depression, respectively, in the guide to the instrument substantiated by a review [40]. The PHQ-9 has been shown to have good psychometric properties [46].

A number of additional measures were administered to assess different aspects of the participants’ symptomatology and experience during the treatments. The 16-item Quick Inventory of Depressive Symptomatology Self-Report (QIDS-16-SR) [47,48] was used in addition to the PHQ-9 because it is a promising questionnaire for assessing depressive symptoms, especially in specialized mental health care and to conduct secondary analyses of primary latent construct of interest: depression. To measure the participants’ satisfaction with the treatments, the 8-item Client Satisfaction Questionnaire (CSQ-8) [49,50] was used. The Credibility and Expectancy Questionnaire (CEQ) [51] was used to measure the participants’ expectancy and judgment of credibility of the treatments. Finally, the level of therapeutic alliance was measured using the Working Alliance Inventory-Short Revised (WAI-SR) [52-54] and was rated by both the participants and the clinicians. For further description of the measures used, we refer to Mathiasen et al [36].

### Statistical Analyses

#### Baseline Characteristics

Characteristics of the sample at baseline was described using descriptive statistics and compared across groups using unpaired *t* tests for continuous variables and chi-square tests for categorical variables. If continuous variables violated the assumption of normality, nonparametric tests were used (Kruskal-Wallis/Wilcoxon signed-rank test). In cases of small cell sizes, exact tests were used (Fisher exact test).

#### Primary Analysis

For the primary analyses a linear multilevel mixed effects model with restricted maximum likelihood estimator was used as intention-to-treat analyses. PHQ-9 scores were used as response variable. Time was included as a fixed effect and as a random effect nested within participant (random slope and intercept) [55]. Correlation between slope and intercept was assumed. All available data were included. Missing values were handled by use of mixed effects models including all available data.

All inferences assumed normally distributed error terms and heteroscedasticity, which were substantiated by visual inspection of a q-q normality plot and a plot of fitted values versus standardized residuals.

Remission was defined as a score of <5 on the PHQ-9. Response to treatment was defined as 50% or more reduction on the PHQ-9.

The noninferiority margin was set to *d*=0.2.

#### Acceptability

Acceptability was estimated from measures of client satisfaction (CSQ-8) and working alliance as reported by the participants (WAI-SR and Technical Alliance Inventory [TAI]) and the clinicians (WAIc). Means were compared across groups using unpaired *t* tests on raw scores using case-wise deletion in case of missing data.

#### Predictor Analyses

Mixed effects models using all available data were applied for analyses of interactions between group and baseline variables by the intention-to-treat principle. One model per predictor was used with PHQ-9 as the response variable in a series of univariate analyses. This was done to test whether baseline characteristics affected outcome differently in the 2 treatments. Inclusion of all parameters would have overfitted the model due to sample size. Time was included as both a fixed effect and a random effect nested in individuals (similar to the primary analysis).

Second, analyses of predictors of symptomatic change in the total sample were also conducted using a mixed effects model with PHQ-9 as response variable. Both multivariate and a series of univariate analyses were conducted. No group interaction was included in these analyses.

#### Completion

Having completed 9 or more (75%) sessions (out of 12) was counted as completion and mean completion rates were compared between groups by unpaired *t* test. The completion rate of the B-CBT group included the sum of online modules and FtF sessions attended.

To assess the odds of noncompletion predicted from the participants’ baseline characteristics, a multivariate logistic regression analysis was conducted. As the response variable, a dichotomous variable for completion was used. Additionally, univariate logistic regression analyses were conducted using 1 model per predictor to investigate whether noncompletion was predicted differently between the FtF-CBT treatment and the B-CBT, which included an interaction term with group.

All calculations were performed using R version 3.4.4 (R Foundation for Statistical Computing) [56]. Mixed effects linear models were calculated using the ImerTest package [57], which fits models by use of the lme4 package [58] and provides *P* values by use of the Satterthwaite degrees of freedom method. Two-way analyses were used with *P*<.05 as the threshold for significance for inferential statistics. All CIs were calculated by bootstrapping using boot.ci [57].

## Results

### Description of Participants

**Figure 1:**
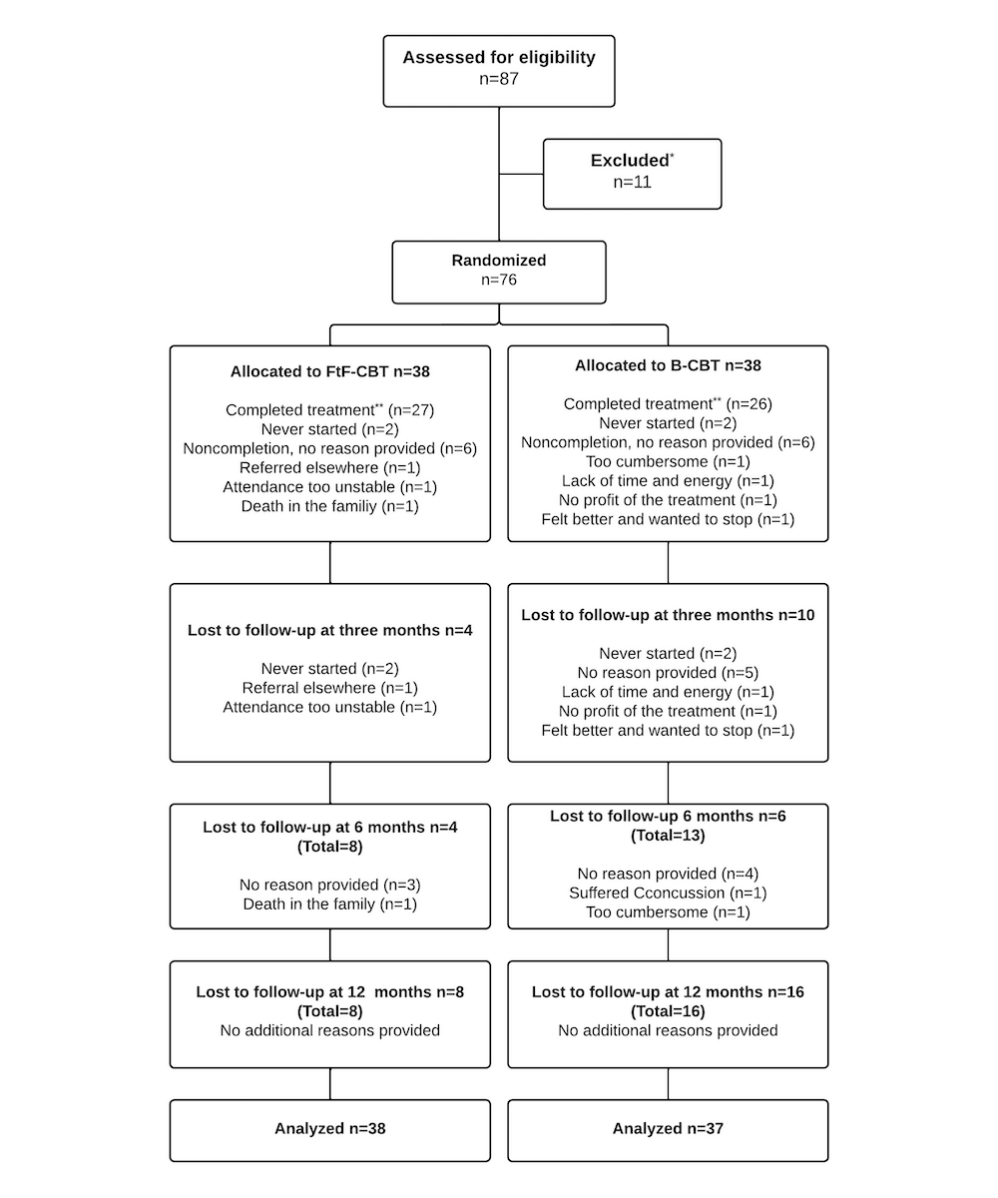
Patient flow. *We did not store any data on any patients who had not provided informed consent. Consequently, no reasons can be provided for this category. **Treatment was regarded as completed when more than 9 sessions were completed. B-CBT: blended cognitive behavioral therapy; FtF-CBT: face-to-face cognitive behavioral therapy.

Table 2 shows the baseline characteristics of all participants and Figure 1 shows the patient flow. In total, 76 were randomized, with 38 allocated to each group. Attrition was somewhat unevenly distributed between groups with 8 being lost to follow-up in the FtF-CBT group and 16 in the B-CBT group. Nonetheless, due to the weekly measurement scheme and the use of mixed effects models, all but one was included in the primary analyses.

The included sample was predominantly female (56/76, 74%) and young with a mean age of 35.0 (SD 13.96) years (median 30 years), although a large age range was seen (18-71 years). Most had moderate to highly severe levels of depression (66/76, 87%) with a mean score of 15.25 (SD 4.04) on the PHQ-9.

No significant differences were observed between the 2 groups on baseline characteristics except for scores on the CEQ measuring the participants’ expectations and credibility of the treatments (see Table 2 for *P* values). The participants in the B-CBT group scored lower on treatment credibility and expectancy of treatment outcome. This raised suspicion as to whether the difference could have been caused by the participants being aware of their group allocation prior to responding to the questionnaire. However, when investigated, it did not seem to be the case. Likewise, no obvious outliers were driving the difference and the distribution of scores seemed reasonable upon visual inspection. A sensitivity analysis of the primary analysis was conducted controlling for the credibility and expectancy scores, but it did not change the outcome.

Among the included sample, 7 were on the brink of violating exclusion criteria, 3 were in psychological treatment at the point of assessment, 2 had some obsessive compulsive disorder symptoms, and 2 were not depressed according to MINI, but scored 9 and 17 on the PHQ-9, respectively. When comparing analyses including or excluding these cases, the outcome did not change. To avoid causing any changes to the analysis plan, all analyses were performed including these participants.

**Table 2:** Characteristics of participants (N=76)^a^.

Characteristics		FtF-CBT^b^	B-CBT^c^	*P* value
**Baseline description**				
	Age, mean (SD)	35.16 (14.14)	34.78 (13.98)	.91
	Female gender, n/N (%)	29/37 (78)	27/37 (73)	.79
	PHQ-9^d^, mean (SD)	16.05 (3.83)	14.42 (4.14)	.08
	Credibility, mean (SD)	0.67 (2.01)	–0.69 (2.28)	.009^e^
	Expectancy, mean (SD)	0.70 (2.22)	–0.72 (2.88)	.02^f^
**Marital status, n/N (%)**				
	Single	13/37 (35)	14/37 (38)	
	Divorced	5/37 (14)	6/37 (16)	
	Widow/widower	0/37 (0)	0/37 (0)	
	Cohabiting	9/37 (24)	8/37 (22)	
	Married	10/37 (27)	8/37 (22)	
	Prefer not to answer	0/37 (0.0)	1/37 (3)	
**Highest education, n/N (%)**				
	Further education <3 years	7/37 (19)	8/37 (22)	
	Further education 3-4 years	13/37 (35)	13/37 (35)	
	Higher education >4 years	4/37 (11)	3/37 (8)	
	Fundamental school <8 years	0/37 (0)	0/37 (0)	
	Fundamental school 9-10 years	3/37 (8)	3/37 (8)	
	Gymnasium (3 years)	9/37 (24)	5/37 (14)	
	Skilled worker	1/37 (3)	5/37 (14)	
**Employment status, n/N (%)**				.34
	Full-time employed	9/36 (25)	4/34 (12)	
	Part-time employed	5/36 (14)	9/34 (27)	
	Sick leave	11/36 (31)	9/34 (27)	
	Leave of absence	2/36 (6)	0/34 (0)	
	Retired	1/36 (3)	1/34 (3)	
	Unemployed	8/36 (22)	11/34 (32)	
**Treatment preference, n/N (%)**				.82
	No preference	16/37 (43)	18/36 (50)	
	Blended care	9/37 (24)	7/36 (19)	
	Face-to-face	12/37 (32)	11/36 (31)	
**Depression severity, n/N (%)**				
	No	0/37 (0)	0/36 (0)	
	Mild	3/37 (8)	4/36 (11)	
	Moderate	9/37 (24)	14/36 (39)	
	Severe	19/37 (51)	16/36 (44)	
	Highly severe	6/37 (16)	2/36 (6)	

^a^Percentages calculated considering attrition.

^b^FtF-CBT: face-to-face cognitive behavioral therapy.

^c^B-CBT: blended cognitive behavioral therapy.

^d^PHQ-9: 9-item Patient Health Questionnaire.

^e^*P*<.01.

^f^*P*<.05.

### Treatment Effect

#### Overview

**Figure 2:**
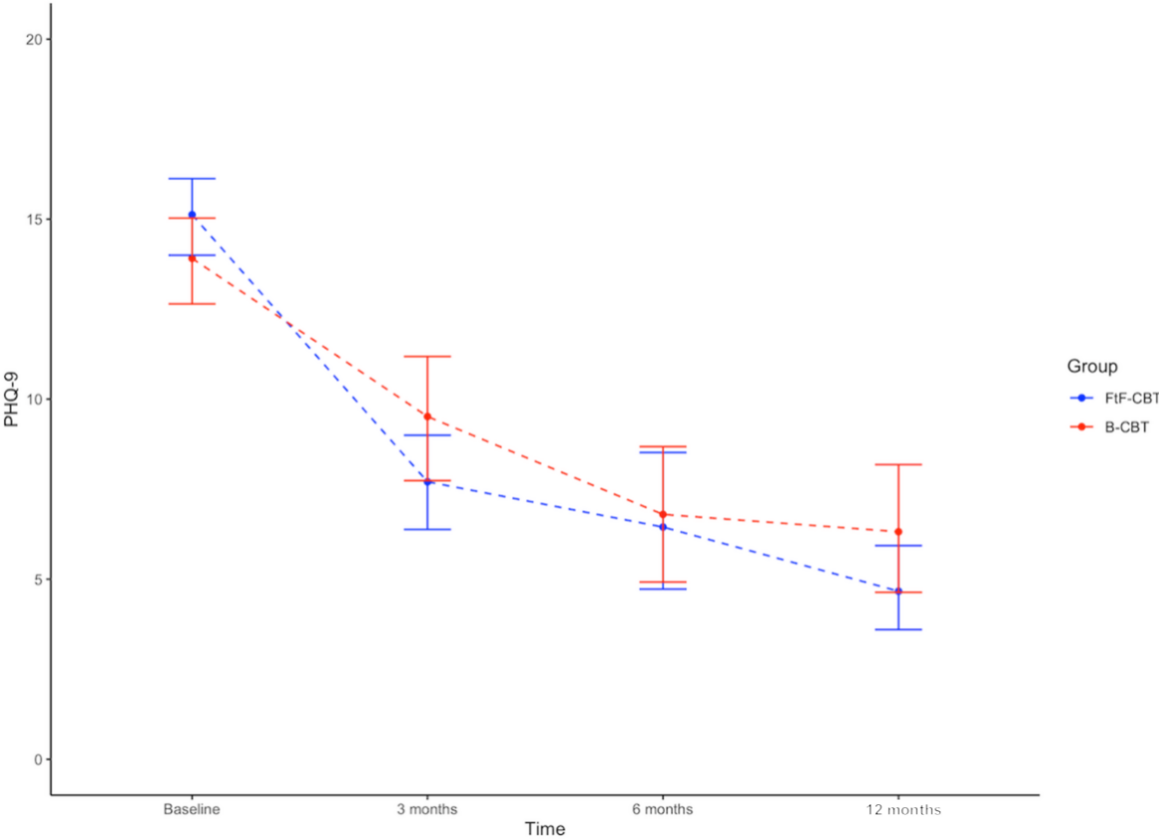
Change in depression on PHQ-9. B-CBT: blended cognitive behavioral therapy; FtF-CBT: face-to-face cognitive behavioral therapy; PHQ-9: 9-item Patient Health Questionnaire.

Initially, we report observed means (Figure 2), standard errors (SEs), and standardized mean differences (Cohen *d*) on the primary outcome measure (PHQ-9). Following this we report results of the mixed effects models.

#### Observed Means

In both groups, large changes in the mean scores within groups were observed on the primary outcome measure (PHQ-9; Table 3). Within the FtF-CBT group, the mean score decreased from 16.05 (SE 0.63) at baseline to 4.67 (SE 0.62) at 12 months’ follow-up. Likewise, in the B-CBT group, the mean score reduced from 14.42 (SE 0.69) to 6.32 (SE 0.95). In both groups the within-group changes in mean scores from baseline to 12-month follow-up revealed large, standardized effect sizes (*d*_FtF-CBT_=–2.04, *d*_B-CBT_=–1.57) [58-61].

**Figure 3:**
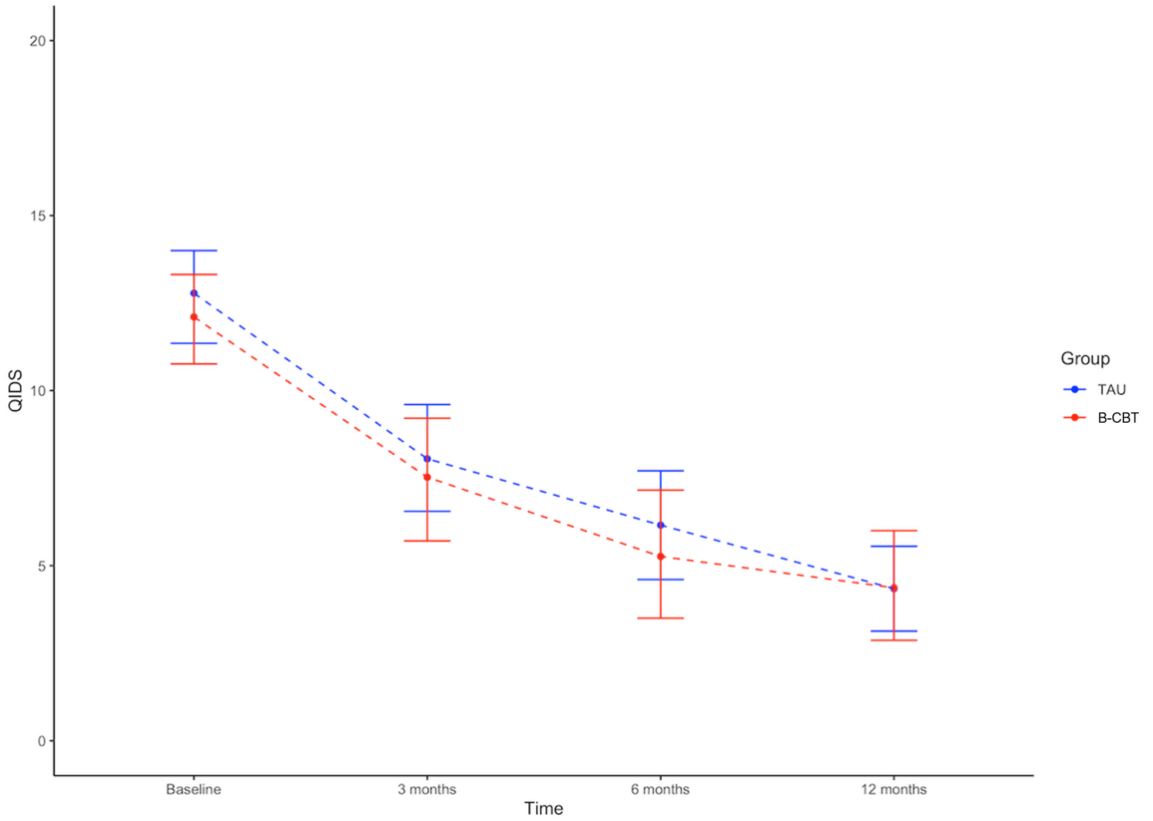
Change in depression on QIDS. B-CBT: blended cognitive behavioral therapy; QIDS: Quick Inventory of Depressive Symptomatology; TAU: treatment as usual.

Between groups, a trend in effect size was noted favoring the FtF-CBT group at 3 months’ follow-up (*d*=–0.5, CI –1.62 to 0.62) but not at 6 months (*d*=0.03, CI –1.43 to 1.49), where the difference had all but disappeared, amounting to just 0.17 points on the PHQ-9 and stayed well within the noninferiority margin of *d*=0.2. At 12 months’ follow-up, a difference could be observed slightly favoring FtF-CBT (*d*=–.42, CI –1.49 to 0.65). However, at all measurement points, the CIs were overlapping and were stretching beyond the noninferiority margin, rendering it impossible to infer generalizability of the results of noninferiority. A similar picture was seen on the secondary outcome of the QIDS (Figure 3).

**Table 3:** Observed means for PHQ-9^a^.

Timepoint		FtF-CBT^b^		B-CBT^c^	
		Mean (SE^d^)	*d* (CI)	Mean (SE)	*d* (CI)
**Baseline**					
	Mean	16.05 (0.63)		14.42 (0.69)	
**Three months**					
	Mean	7.71 (0.7)		9.93 (0.92)	
	Between-groups effect size^e^		–0.5 (–1.62 to –1.17)		
	Within-group effect size^f^		–2.04 (–2.91 to –1.17)		–1.57 (–2.68 to –0.46)
**Six months**					
	Mean	6.97 (1.09)		6.8 (0.99)	
	Between-groups effect size^e^		0.03 (–1.43 to 1.49)		
	Within-group effect size^f^		–2.09 (–3.29 to –0.89)		–1.52 (–2.67 to –0.37)
**Twelve months**					
	Mean	4.67 (0.62)		6.32 (0.95)	
	Between-groups effect size^e^		–0.42 (–1.49 to 0.65)		
	Within-group effect size^f^		–2.04 (-2.91 to –1.17)		–1.57 (–2.68 to –0.46)

^a^PHQ-9: 9-item Patient Health Questionnaire.

^b^FtF-CBT: face-to-face cognitive behavioral therapy.

^c^B-CBT: blended cognitive behavioral therapy.

^d^SE: standard error.

^e^Independent samples [60].

^f^Formula 3 in Dunlap et al [61] for dependent samples.

#### Primary Analyses

As can be seen in Table 4, the primary analyses using linear mixed effects models with the PHQ-9 as outcome variable confirmed the within-group improvements in both groups being significant (FtF-CBT: β=–.03; SE 0.00; *P*<.001 and B-CBT: β=–.02; SE 0.00; *P*<.001), which was also the case for the QIDS scores (FtF-CBT: β=–.02; SE 0.00; *P*<.001 and B-CBT: β=–.01; SE 0.00; *P*<.001). The β values are small, as they represent the change in the outcome measure per day. Between groups, a very small but significant interaction effect was observed on the PHQ-9 (β=.01; SE 0.00; *P*=.03), indicating a slight advantage of the FtF-CBT group. However, this was not the case on the QIDS (β=.01; SE 0.00; *P*=.05), which was just above the significance level.

A negative correlation was observed between intercept and slope in the primary model (*r*=–0.29), indicating that a higher initial score (intercept) correlated with a steeper negative slope (symptomatic improvement).

**Table 4:** Results of the mixed effects linear regressions.

Fixed effects	Primary analysis			Within-group FtF-CBT^a^			Within-group B-CBT^b^			
	Estimates	SE^c^	*P* value	Estimates	SE	*P* value	Estimates	SE	*P* value	
(Intercept)	12.71	0.61	<2 × 10^–16d^	12.71	0.60	2 × 10^–16d^	12.47	0.64	<2 × 10^–16d^	
Time	–0.03	0.00	3.12 × 10^–16d^	–0.03	0.00	2.24 × 10^–11d^	–0.02	0.00	3.59 × 10^–9d^	
Group B-CBT	–0.23	0.89	.80	N/A^e^	N/A	N/A	N/A	N/A	N/A	
Time × group B-CBT	0.01	0.00	.03^f^	N/A	N/A	N/A	N/A	N/A	N/A	

^a^FtF-CBT: face-to-face cognitive behavioral therapy.

^b^B-CBT: blended cognitive behavioral therapy.

^c^SE: standard error.

^d^*P*<.001.

^e^N/A: not applicable.

^f^*P*<.05.

#### Acceptability

There was no significant difference in client satisfaction between groups (mean difference –2.18; t_39.36_=2.16; *P*=.15). Furthermore, no significant difference in working alliance was observed when reported by the participants (mean difference 2.31; t_50.08_=1.14; *P*=.26). However, the difference between groups was larger and significant when rated by the treating clinicians (mean difference 6.27; t_58.51_=3.68; *P*<.001).

The rates of participants responding to treatment at 12 months based on the PHQ-9 were 83% (25/30) in the FtF-CBT group and 64% (14/22) in the B-CBT group. The remission rates at 12 months were 60% (18/30) for the FtF-CBT group and 50% (11/22) for the B-CBT group. When inspecting all individual slopes of the primary model, we found no negative individual slopes, indicating that none of the participants’ depressive condition deteriorated.

Finally, 20 randomly selected audio-recorded sessions were examined for treatment fidelity by an external expert in clinical psychology. Among the sample, session numbers ranged from 3 to 12, 3 of 4 therapists were represented, and both groups were well represented, with 14 sessions being from the FtF-CBT group. The mean score of treatment fidelity was 4.25 (SD 0.71) on a scale ranging from 1 (not compliant with the protocol) to 5 (completely compliant with the protocol).

#### Predictor Analyses

In a multivariate analysis of the total sample, only being on sick leave and preferring blended care predicted outcome. Being on sick leave added to the slope (3.96; SE 1.54; *P*=.02), that is, produced a smaller reduction in symptom change. Preferring blended care subtracted from the slope estimate (–3.25; SE 1.53; *P*=.04), thus signifying an increase in symptom reduction. Table 5 summarizes all predictor variables with SEs and *P* values from a multivariate analysis of the total sample.

In a series of univariate interaction analyses of each parameter × group, there was a significant interaction effect of being part-time employed (β=–5.83; SE 2.68; *P*=.03) or unemployed (β=–7.59; SE 2.52; *P*=.004), with both favoring B-CBT.

**Table 5:** Predictor analysis.

Variables		Estimate	SE^a^	*P* value
(Intercept)		16.85	4.11	<.001
Time		–0.02	0.00	<.001
Age		0.00	0.07	.98
Female sex		–0.51	0.07	.71
**Marital status**				
	Divorced	–0.98	2.07	.64
	Cohabiting	0.23	1.39	.87
	Married	–4.08	2.06	.06
	No answer	–1.41	3.98	.73
**Highest education**				
	Further education 3-4 years	–1.76	1.45	.23
	Higher education > 4 years	–3.08	1.76	.09
	Fundamental school 9-10 years	–2.85	3.14	.37
	High school (3 years)	–3.41	1.76	.06
	Skilled worker	–0.80	2.55	.76
**Employment status**				
	Part-time employed	1.09	1.70	.53
	Sick leave	3.96	1.54	.02^b^
	Leave of absence	1.48	3.12	.64
	Retired	–0.53	3.48	.88
	Unemployed	0.26	1.63	.87
**Preference and expectancy**				
	Blended care	–3.25	1.53	.04^b^
	Face-to-face	–1.18	1.28	.36
	Credibility	0.24	0.31	.45
	Expectancy	–0.37	0.28	.20
**Usability**				
	System usability	–0.05	0.11	.73

^a^SE: standard error.

^b^*P*<.05.

#### Completion

In total, 53 (70%) completed the treatment; 27 (71%) from the FtF-CBT group and 26 (68%) from the B-CBT group. Completers as well as noncompleters showed a significant effect of time (completers: β=–.03, *P*<.001 and noncompleters: β=–.03, *P*<.001). In an analysis of the total sample including a binary interaction term for completion, no significant interaction was seen (β=.00, *P*=.43), which indicated that there was no difference in effect between completers and noncompleters.

In the FtF-CBT group, a mean of 9.8 sessions was completed. In the B-CBT group, a mean of 9.2 sessions was completed. The mean difference was not significant (t_74_=–0.70, *P*=.49). Table 6 presents the reasons for noncompletion.

We did not find any variables that significantly predicted noncompletion in multivariate analyses of the total sample nor did we find any interaction effect between any of the baseline characteristics and groups in a series of univariate analyses, indicating no difference in risk of noncompletion on any baseline characteristic between groups.

**Table 6:** Reasons for noncompletion (n=15).

Reasons for noncompletion	Value, *n*
Inactive	2
No reason given	7
Felt it was too strenuous	1
Referred to other treatment	2
Wished to end the treatment	2
Felt unable to profit from the treatment	1

## Discussion

### Principal Findings

The main aim of this study was to compare the clinical effectiveness of B-CBT with traditional FtF-CBT, because the blended format may hold the promise to combine advantages of the traditional and the new format of delivery. In this study, we found very similar trajectories of improvement in both groups as well as on measures of other parameters, such as working alliance and retention. However, it was possible to detect a significant difference between groups in slight favor of FtF-CBT.

The sample corresponded well with what is seen among patients with depression in the primary health care sector in Denmark regarding gender and age distribution [3,62]. Further, the distribution of the highest education level resembles that of the general Danish population [63].

The mean symptomatic change observed in the B-CBT condition closely approximated that of the FtF-CBT group. This is in line with meta-analyses of guided iCBT for depression [16,64] and exceeded what has been observed at the clinic “Internetpsykiatrien,” from which this study recruited [17]. It also aligned well with the large effect (*d*=1.08) seen in the study of B-CBT by Lungu et al [33]. Nonetheless, an interaction effect could be detected between the 2 groups favoring FtF-CBT, although the effect was very small. This effect seems to be driven by the FtF group experiencing a faster symptom reduction during treatment, an effect that disappeared at 6 months’ follow-up. However, at 12 months’ follow-up, the FtF group showed a larger reduction compared with B-CBT. It is important to note, though, that none of these differences were statistically significant, thus a difference between groups cannot be inferred. Unfortunately, with the variance observed, it is not possible to infer noninferiority either (a difference no bigger than *d*=.02), due to the CIs stretching beyond the noninferiority margin. Consequently, although promising, the study is inconclusive regarding noninferiority but may support no superiority of either treatment.

The working alliance between the patient and clinician has often been argued to be one of the most important nonspecific factors of psychotherapy [65]. It is, therefore, very interesting that although half of the sessions in B-CBT were computerized, the therapeutic alliance was rated equally well in both groups. There was a tendency among the clinicians to rate it higher in the FtF group, but the difference was not significant. Similar findings are also emerging in other studies of blended care [66]. Furthermore, these studies are starting to point to details differing between B-CBT and FtF-CBT, for example, therapist ratings sometimes correlating more with treatment outcome than patient ratings conflicting with research on the working alliance in FtF-CBT [65]. Further study detailing the dynamics of the therapeutic alliance in B-CBT and the difference between that and FtF-CBT is needed.

Acceptability of the blended format seemed to be high as judged by levels of client satisfaction and working alliance, where no significant differences were observed. Furthermore, high retention rates among participants and high treatment fidelity rates for the clinicians indicated satisfaction and acceptability with the treatment.

As is commonly found [67-69], a negative correlation between intercept and slope was seen, indicating that a higher baseline severity of depression was associated with larger symptom reduction.

Interestingly, 1 variable was able to distinguish between the 2 groups in predicting outcome differently. An interaction effect was observed between employment status and group. Being part-time employed or unemployed both favored the blended care group. We speculate that this may be due to the B-CBT treatment always being available, possibly increasing the chance of treatment engagement if the participant has more free time. Consequently, this is a potential candidate variable for stratification of treatment or a prescriptive variable. In a different approach comparing variables predicting outcomes separately for the 2 groups in a larger sample across 4 countries [70], a lower quality of life and being widowed predicted lower treatment outcome in the blended condition. However, this approach does not include the parameters in a single model comparing the conditions directly in an interaction term. Nonetheless, these findings are encouraging and prompt the need to further study potential variables for stratification of patients.

This study is well aligned with previous observations of completion rates in both guided iCBT and traditional CBT [71-74]. The reasons provided by either therapists or patients for treatment dropout varied and there were too few to differentiate between the groups.

In disagreement with what has previously been found in guided iCBT, we observed that no baseline characteristics predicted noncompletion [25,75,76]. It may be speculated to be caused by the increased therapist contact in the blended format, which may serve as a protective factor against noncompletion.

### Limitations

This study compared directly the formats of delivery with a minimum of the variance explained by differences in therapeutic methods, which is both a strength and a weakness of the design. While it lends itself well to compare the 2 treatment formats, it also somewhat limits the ecological validity, making it more difficult to make inferences about the clinical effect in routine care. Furthermore, because the study recruited from Internetpsykiatrien, which offers self-referral, even though the clinic is situated in secondary care, it can be difficult to generalize to future implementations. Additionally, only the B-CBT group received reminders about homework assignments. This might be a confounder, for example, there is a risk participants in this group grew weary of the reminders, thus affecting the perception of the treatment negatively. Finally, due to the small sample size, we had difficulty inferring noninferiority, although the many observations and advanced statistical procedures appear to have compensated for that to some degree. The large EU study E-COMPARED will be able to pool data from many studies, including this one, and may thus be able to reach more robust conclusions about noninferiority.

### Conclusions

In this study, feasibility of B-CBT was demonstrated as well as large and significant within-group effect sizes were produced. In fact, it was seen that practically without loss of treatment effect, completion rates, and therapeutic alliance, it was possible to substitute half of the FtF consultations with online modules when treating adult depression. This is remarkable and lends support to the hypothesis of noninferiority of B-CBT and should lead to the further study of this promising treatment format. However, it should also be noted that small differences were observed favoring the FtF-CBT group. Although not significant, it may be that FtF treatment works faster, and has a better long-term effect for some patients. The results, therefore, need to be replicated in larger samples or with pooled data from multicenter trials as will be done in the E-COMPARED study. Additionally, further studies should explore the applicability of B-CBT in different patient populations and clinical settings. Furthermore, participants’ digital health literacy should be measured in future studies.

## Appendix

Multimedia Appendix 1 CONSORT-eHEALTH checklist (V 1.6.1).

## Abbreviations

B-CBT: blended cognitive behavioral therapy
BDI-II: Beck Depression Inventory II
CBT: cognitive behavioral therapy
CEQ: Credibility and Expectancy Questionnaire
CSQ-8: 8-item Client Satisfaction Questionnaire
DSM-IV-TR: Diagnostic and Statistical Manual of Mental disorders 4th edition text revision
ENTER: e-Mental Health Research
EU: European Union
FtF-CBT: face-to-face cognitive behavioral therapy
M.I.N.I.: Mini-International Neuropsychiatric Interview
OPEN: Odense Patient data Exploratory Network
PHQ-9: 9-item Patient Health Questionnaire
QIDS-16-SR: 16-item Quick Inventory of Depressive Symptomatology Self-Report
SE: standard error
TAI: Technical Alliance Inventory
TAU: treatment as usual
WAI: Working Alliance Inventory-Short Revised
WHO: World Health Organization
