# Supplementary material for: The Clinical Effectiveness of Blended Cognitive Behavioral Therapy Compared With Face-to-Face Cognitive Behavioral Therapy for Adult Depression: Randomized Controlled Noninferiority Trial
Source: J Med Internet Res. 2022 Sep 7;24(9):e36577. doi: 10.2196/36577 (PMC9543221; doi:10.2196/36577)
Supplement: Multimedia Appendix 1 [file jmir_v24i9e36577_app1.pdf]

CONSORT-EHEALTH (V 1.6.1) - Submission/Publication Form

The CONSORT-EHEALTH checklist is intended for authors of randomized trials evaluating web-based and Internet-based applications/interventions, including mobile interventions, electronic games (incl multiplayer games), social media, certain telehealth applications, and other interactive and/or networked electronic applications. Some of the items (e.g. all subitems under item 5 - description of the intervention) may also be applicable for other study designs.

The goal of the CONSORT EHEALTH checklist and guideline is to be
a) a guide for reporting for authors of RCTs,
b) to form a basis for appraisal of an ehealth trial (in terms of validity)

CONSORT-EHEALTH items/subitems are MANDATORY reporting items for studies published in the Journal of Medical Internet Research and other journals / scientific societies endorsing the checklist.

Items numbered 1., 2., 3., 4a., 4b etc are original CONSORT or CONSORT-NPT (non-pharmacologic treatment) items.
Items with Roman numerals (i., ii, iii, iv etc.) are CONSORT-EHEALTH extensions/clarifications.

As the CONSORT-EHEALTH checklist is still considered in a formative stage, we would ask that you also RATE ON A SCALE OF 1-5 how important/useful you feel each item is FOR THE PURPOSE OF THE CHECKLIST and reporting guideline (optional).

Mandatory reporting items are marked with a red *.
In the textboxes, either copy & paste the relevant sections from your manuscript into this form - please include any quotes from your manuscript in QUOTATION MARKS,
or answer directly by providing additional information not in the manuscript, or elaborating on why the item was not relevant for this study.

YOUR ANSWERS WILL BE PUBLISHED AS A SUPPLEMENTARY FILE TO YOUR PUBLICATION IN JMIR AND ARE CONSIDERED PART OF YOUR PUBLICATION (IF ACCEPTED).
Please fill in these questions diligently. Information will not be copyedited, so please use proper spelling and grammar, use correct capitalization, and avoid abbreviations.

DO NOT FORGET TO SAVE AS PDF _AND_ CLICK THE SUBMIT BUTTON SO YOUR ANSWERS ARE IN OUR DATABASE !!!

Citation Suggestion (if you append the pdf as Appendix we suggest to cite this paper in the caption):
Eysenbach G, CONSORT-EHEALTH Group
CONSORT-EHEALTH: Improving and Standardizing Evaluation Reports of Web-based and Mobile Health Interventions
J Med Internet Res 2011;13(4):e126
URL: http://www.jmir.org/2011/4/e126/
doi: 10.2196/jmir.1923
PMID: 22209829

Log ind på Google for at gemme dine data. Få flere oplysninger

*Skal udfyldes

Your name *

First Last

Kim Mathiasen

Primary Affiliation (short), City, Country *

University of Toronto, Toronto, Canada

University of Southern Denmark, Odense Denm

Your e-mail address *

abc@gmail.com

kmathiasen@health.sdu.dk

Title of your manuscript *

Provide the (draft) title of your manuscript.

Clinical Effectiveness of Blended CBT Compared vs Face-to-Face CBT for adult depression: a Randomised Controlled Non-Inferiority Trial

Name of your App/Software/Intervention *

If there is a short and a long/alternate name, write the short name first and add the long name in brackets.

NoDep

Evaluated Version (if any)

e.g. "V1", "Release 2017-03-01", "Version 2.0.27913"

Dit svar

Language(s) *

What language is the intervention/app in? If multiple languages are available, separate by comma (e.g. "English, French")

Danish

URL of your Intervention Website or App

e.g. a direct link to the mobile app on app in appstore (itunes, Google Play), or URL of the website. If the intervention is a DVD or hardware, you can also link to an Amazon page.

Dit svar

URL of an image/screenshot (optional)

Dit svar

Accessibility *

Can an enduser access the intervention presently?

- ○ access is free and open
- ○ access only for special usergroups, not open
- ○ access is open to everyone, but requires payment/subscription/in-app purchases
- ○ app/intervention no longer accessible
- ◉ Andet: The program content has since been updated and moved to a differer

Primary Medical Indication/Disease/Condition *

e.g. "Stress", "Diabetes", or define the target group in brackets after the condition, e.g. "Autism (Parents of children with)", "Alzheimers (Informal Caregivers of)"

Depression

Primary Outcomes measured in trial *

comma-separated list of primary outcomes reported in the trial

The Patient Health Questionnaire-9 (PHQ-9)

Secondary/other outcomes

Are there any other outcomes the intervention is expected to affect?

The Quick Inventory of Depressive Symptomatology Self-Report (QIDS-16-SR), the Client Satisfaction Questionnaire (CSQ-8), The Credibility and Expectancy Questionnaire (CEQ), the Working Alliance Inventory (WAI-SR)

Recommended "Dose" *

What do the instructions for users say on how often the app should be used?

- (x) Approximately Daily
- ( ) Approximately Weekly
- ( ) Approximately Monthly
- ( ) Approximately Yearly
- ( ) "as needed"
- ( ) Andet:

Approx. Percentage of Users (starters) still using the app as recommended after 3 months *

- ( ) unknown / not evaluated
- ( ) 0-10%
- ( ) 11-20%
- ( ) 21-30%
- ( ) 31-40%
- ( ) 41-50%
- ( ) 51-60%
- ( ) 61-70%
- ( ) 71%-80%
- (x) 81-90%
- ( ) 91-100%
- ( ) Andet:

Overall, was the app/intervention effective? *

- ( ) yes: all primary outcomes were significantly better in intervention group vs control
- ( ) partly: SOME primary outcomes were significantly better in intervention group vs control
- ( ) no statistically significant difference between control and intervention
- ( ) potentially harmful: control was significantly better than intervention in one or more outcomes
- ( ) inconclusive: more research is needed
- (x) Andet: As this was a non-inferiority study, the results warrants a slightly diffe

Article Preparation Status/Stage *

At which stage in your article preparation are you currently (at the time you fill in this form)

- ○ not submitted yet - in early draft status
- ○ not submitted yet - in late draft status, just before submission
- ◉ submitted to a journal but not reviewed yet
- ○ submitted to a journal and after receiving initial reviewer comments
- ○ submitted to a journal and accepted, but not published yet
- ○ published
- ○ Andet:

Journal *

If you already know where you will submit this paper (or if it is already submitted), please provide the journal name (if it is not JMIR, provide the journal name under "other")

- ○ not submitted yet / unclear where I will submit this
- ◉ Journal of Medical Internet Research (JMIR)
- ○ JMIR mHealth and UHealth
- ○ JMIR Serious Games
- ○ JMIR Mental Health
- ○ JMIR Public Health
- ○ JMIR Formative Research
- ○ Other JMIR sister journal
- ○ Andet:

Is this a full powered effectiveness trial or a pilot/feasibility trial? *

- ○ Pilot/feasibility
- ◉ Fully powered

Manuscript tracking number *

If this is a JMIR submission, please provide the manuscript tracking number under "other" (The ms tracking number can be found in the submission acknowledgement email, or when you login as author in JMIR. If the paper is already published in JMIR, then the ms tracking number is the four-digit number at the end of the DOI, to be found at the bottom of each published article in JMIR)

- ○ no ms number (yet) / not (yet) submitted to / published in JMIR
- ◉ Andet: 36577

TITLE AND ABSTRACT

1a) TITLE: Identification as a randomized trial in the title

1a) Does your paper address CONSORT item 1a? *

I.e does the title contain the phrase "Randomized Controlled Trial"? (if not, explain the reason under "other")

- ◉ yes
- ○ Andet:

1a-i) Identify the mode of delivery in the title

Identify the mode of delivery. Preferably use “web-based” and/or “mobile” and/or “electronic game” in the title. Avoid ambiguous terms like “online”, “virtual”, “interactive”. Use “Internet-based” only if Intervention includes non-web-based Internet components (e.g. email), use “computer-based” or “electronic” only if offline products are used. Use “virtual” only in the context of “virtual reality” (3-D worlds). Use “online” only in the context of “online support groups”. Complement or substitute product names with broader terms for the class of products (such as “mobile” or “smart phone” instead of “iphone”), especially if the application runs on different platforms.

	1	2	3	4	5	
subitem not at all important	○	○	○	◉	○	essential

Ryd markering

Does your paper address subitem 1a-i? *

Copy and paste relevant sections from manuscript title (include quotes in quotation marks "like this" to indicate direct quotes from your manuscript), or elaborate on this item by providing additional information not in the ms, or briefly explain why the item is not applicable/relevant for your study

"Blended CBT Compared to Face-to-Face CBT"

1a-ii) Non-web-based components or important co-interventions in title

Mention non-web-based components or important co-interventions in title, if any (e.g., "with telephone support").

	1	2	3	4	5	
subitem not at all important	○	○	○	●	○	essential

Ryd markering

Does your paper address subitem 1a-ii?

Copy and paste relevant sections from manuscript title (include quotes in quotation marks "like this" to indicate direct quotes from your manuscript), or elaborate on this item by providing additional information not in the ms, or briefly explain why the item is not applicable/relevant for your study

"Blended CBT Compared to Face-to-Face CBT"

1a-iii) Primary condition or target group in the title

Mention primary condition or target group in the title, if any (e.g., "for children with Type I Diabetes")
Example: A Web-based and Mobile Intervention with Telephone Support for Children with Type I Diabetes: Randomized Controlled Trial

	1	2	3	4	5	
subitem not at all important	○	○	○	●	○	essential

Ryd markering

Does your paper address subitem 1a-iii? *

Copy and paste relevant sections from manuscript title (include quotes in quotation marks "like this" to indicate direct quotes from your manuscript), or elaborate on this item by providing additional information not in the ms, or briefly explain why the item is not applicable/relevant for your study

"Blended CBT Compared vs Face-to-Face CBT for adult depression"

1b) ABSTRACT: Structured summary of trial design, methods, results, and conclusions

NPT extension: Description of experimental treatment, comparator, care providers, centers, and blinding status.

1b-i) Key features/functionalities/components of the intervention and comparator in the METHODS section of the ABSTRACT

Mention key features/functionalities/components of the intervention and comparator in the abstract. If possible, also mention theories and principles used for designing the site. Keep in mind the needs of systematic reviewers and indexers by including important synonyms. (Note: Only report in the abstract what the main paper is reporting. If this information is missing from the main body of text, consider adding it)

	1	2	3	4	5	
subitem not at all important	○	○	○	◉	○	essential

Ryd markering

Does your paper address subitem 1b-i? *

Copy and paste relevant sections from the manuscript abstract (include quotes in quotation marks "like this" to indicate direct quotes from your manuscript), or elaborate on this item by providing additional information not in the ms, or briefly explain why the item is not applicable/relevant for your study

"B-CBT comprised six sessions of FtF-CBT alternated with six to eight web-based CBT self-help modules. TAU comprised twelve sessions of FtF-CBT"

1b-ii) Level of human involvement in the METHODS section of the ABSTRACT

Clarify the level of human involvement in the abstract, e.g., use phrases like “fully automated” vs. “therapist/nurse/care provider/physician-assisted” (mention number and expertise of providers involved, if any). (Note: Only report in the abstract what the main paper is reporting. If this information is missing from the main body of text, consider adding it)

	1	2	3	4	5	
subitem not at all important	○	○	○	◉	○	essential

Ryd markering

Does your paper address subitem 1b-ii?

Copy and paste relevant sections from the manuscript abstract (include quotes in quotation marks "like this" to indicate direct quotes from your manuscript), or elaborate on this item by providing additional information not in the ms, or briefly explain why the item is not applicable/relevant for your study

"B-CBT comprised six sessions of FtF-CBT alternated with six to eight web-based CBT self-help modules. TAU comprised twelve sessions of FtF-CBT"

1b-iii) Open vs. closed, web-based (self-assessment) vs. face-to-face assessments in the METHODS section of the ABSTRACT

Mention how participants were recruited (online vs. offline), e.g., from an open access website or from a clinic or a closed online user group (closed usergroup trial), and clarify if this was a purely web-based trial, or there were face-to-face components (as part of the intervention or for assessment). Clearly say if outcomes were self-assessed through questionnaires (as common in web-based trials). Note: In traditional offline trials, an open trial (open-label trial) is a type of clinical trial in which both the researchers and participants know which treatment is being administered. To avoid confusion, use “blinded” or “unblinded” to indicated the level of blinding instead of “open”, as “open” in web-based trials usually refers to “open access” (i.e. participants can self-enrol). (Note: Only report in the abstract what the main paper is reporting. If this information is missing from the main body of text, consider adding it)

	1	2	3	4	5	
subitem not at all important	○	○	○	◉	○	essential

Ryd markering

Does your paper address subitem 1b-iii?

Copy and paste relevant sections from the manuscript abstract (include quotes in quotation marks "like this" to indicate direct quotes from your manuscript), or elaborate on this item by providing additional information not in the ms, or briefly explain why the item is not applicable/relevant for your study

"The trial was researcher blinded (unblinded for participants and clinicians)", "...and were recruited via a national iCBT clinic. "

1b-iv) RESULTS section in abstract must contain use data

Report number of participants enrolled/assessed in each group, the use/uptake of the intervention (e.g., attrition/adherence metrics, use over time, number of logins etc.), in addition to primary/secondary outcomes. (Note: Only report in the abstract what the main paper is reporting. If this information is missing from the main body of text, consider adding it)

	1	2	3	4	5	
subitem not at all important	○	○	○	○	◉	essential

Ryd markering

Does your paper address subitem 1b-iv?

Copy and paste relevant sections from the manuscript abstract (include quotes in quotation marks "like this" to indicate direct quotes from your manuscript), or elaborate on this item by providing additional information not in the ms, or briefly explain why the item is not applicable/relevant for your study

"A total of N=76 were randomised, n=38 allocated to each treatment. Age ranged from 18 to 71 years (SD=13.96) with 73.7% (n=56) female. Attrition rate was 19.7% (n=15), less from the FtF-CBT group (15.8%, n=6) than the B-CBT group (23.7%, n=9). 53 (69.7%) completed ≥9 sessions almost equally distributed between the groups (nFtF-CBT=27 (71.1%), nB-CBT= 26 (68.4%)). PHQ-9 reduced 11.38 points in the FtF-CBT group and 8.10 in the B-CBT group. At six months, the mean difference was a mere 0.17 points. The primary analyses confirmed large and significant within group reductions in both groups (FtF-CBT: ß=-0.03, SE=0.00, p<.001; B-CBT: ß=-0.02, SE=0.00, p < .001). A small but significant interaction effect was observed between groups (ß=0.01, SE=0.00, p=.026)."

1b-v) CONCLUSIONS/DISCUSSION in abstract for negative trials

Conclusions/Discussions in abstract for negative trials: Discuss the primary outcome - if the trial is negative (primary outcome not changed), and the intervention was not used, discuss whether negative results are attributable to lack of uptake and discuss reasons. (Note: Only report in the abstract what the main paper is reporting. If this information is missing from the main body of text, consider adding it)

	1	2	3	4	5	
subitem not at all important	○	○	○	◉	○	essential

Ryd markering

Does your paper address subitem 1b-v?

Copy and paste relevant sections from the manuscript abstract (include quotes in quotation marks "like this" to indicate direct quotes from your manuscript), or elaborate on this item by providing additional information not in the ms, or briefly explain why the item is not applicable/relevant for your study

Primary outcome did change

INTRODUCTION

2a) In INTRODUCTION: Scientific background and explanation of rationale

2a-i) Problem and the type of system/solution

Describe the problem and the type of system/solution that is object of the study: intended as stand-alone intervention vs. incorporated in broader health care program? Intended for a particular patient population? Goals of the intervention, e.g., being more cost-effective to other interventions, replace or complement other solutions? (Note: Details about the intervention are provided in “Methods” under 5)

	1	2	3	4	5	
subitem not at all important	○	○	○	○	◉	essential

Ryd markering

Does your paper address subitem 2a-i? *

Copy and paste relevant sections from the manuscript (include quotes in quotation marks "like this" to indicate direct quotes from your manuscript), or elaborate on this item by providing additional information not in the ms, or briefly explain why the item is not applicable/relevant for your study

"Depression is a prevalent and disabling disorder with a high risk of relapse and large individual and societal costs (1–6). Effective treatments do exist (7), although a large gap is seen between the need for - and use of treatments (8). This gap has led researchers to explore alternative modes of treatment delivery. One such novel treatment format is Internet based cognitive behavioural therapy (iCBT) (9–17), in which the patient is administered access to an online treatment programme based on cognitive behavioural therapy. The highest clinical effect is seen when clinical guidance is provided during the course of treatment (18–20). However, despite the evidence for the effect of guided iCBT, there is a need for increased therapist contact among some patient groups as well as a need to provide a treatment format, which is more compatible with, and thus easier to implement in, the existing health care services (21–24).

Combining iCBT with traditional face-to-face consultations in a blended CBT format (B-CBT), in which both online components and face-to-face sessions are included in one coherent CBT protocol, may alleviate some of the difficulties associated with iCBT for depression, while preserving some of the advantages of both iCBT and face-to-face CBT (FtF-CBT) alike. Firstly, by including face-to-face sessions, the therapist can individualise the therapy taking the idiosyncratic case formulation of the patient, the specific disorder, and possible co-morbidity into account. Secondly, since B-CBT in the format tested in the present study only provides half the number of sessions as traditional FtF-CBT, the capacity of the treating clinician is increased compared to traditional CBT. Third, the burden and cost of travel by the patient can be reduced compared to FtF-CBT. Fourth, the online modules are available at the time and place needed by the patients - and they can be re-viewed multiple times. Fifth, the inherently structured format of the online modules ensures high treatment fidelity e.g., by delivering the same psychoeducation and exercises to all patients. sixth, one of the principal barriers for the uptake of iCBT seems to be scepticism concerning allotting the majority of therapy to a computer (25), a barrier possibly alleviated by the blended format of B-CBT (26). Finally, the blended format is more compatible with the existing health care services and as a consequence should be easier to implement than iCBT (27)."

2a-ii) Scientific background, rationale: What is known about the (type of) system

Scientific background, rationale: What is known about the (type of) system that is the object of the study (be sure to discuss the use of similar systems for other conditions/diagnoses, if appropiate), motivation for the study, i.e. what are the reasons for and what is the context for this specific study, from which stakeholder viewpoint is the study performed, potential impact of findings [2]. Briefly justify the choice of the comparator.

	1	2	3	4	5	
subitem not at all important	○	○	○	○	◉	essential

Ryd markering

Does your paper address subitem 2a-ii? *

Copy and paste relevant sections from the manuscript (include quotes in quotation marks "like this" to indicate direct quotes from your manuscript), or elaborate on this item by providing additional information not in the ms, or briefly explain why the item is not applicable/relevant for your study

"Few studies have investigated the use of blended care combining internet based psychotherapeutic modules and face-to-face sessions into one coherent treatment manual to treat adult depression (28–31). Generally, however, they do indicate positive outcomes. In a randomised controlled trial conducted in primary care in Tromsø, Norway clinical psychologists delivered thirty-minutes sessions following each online module (29). They were able to document a significant difference with a moderate to large effect size (d=0.65) on depressive symptoms (BDI-II) favouring blended care over waiting list. The intervention predominantly received positive evaluations suggesting acceptability and satisfaction with the treatment. Additionally, a qualitative study found that the face-to-face consultations increased motivation to persist with the iCBT program (32). Another recent example is the development and initial evaluation of a programme for B-CBT in The Netherlands. This was tested at an outpatient clinic of a specialised mental health care centre in Amsterdam. The study was designed as a feasibility study and included only nine patients. However, the patients perceived the intervention as positive although the authors rightly note that no conclusion can be derived from such a small sample (31)."

2b) In INTRODUCTION: Specific objectives or hypotheses

Does your paper address CONSORT subitem 2b? *

Copy and paste relevant sections from the manuscript (include quotes in quotation marks "like this" to indicate direct quotes from your manuscript), or elaborate on this item by providing additional information not in the ms, or briefly explain why the item is not applicable/relevant for your study

"The primary aim of the present study was to compare the clinical effectiveness of B-CBT for major depressive disorder (MDD) in adults with treatment as usual (TAU) defined as twelve sessions of FtF-CBT. It is hypothesised that B-CBT will be no less clinically effective than FtF-CBT, and that it will be acceptable and satisfactory to patients and clinicians."

METHODS

3a) Description of trial design (such as parallel, factorial) including allocation ratio

Does your paper address CONSORT subitem 3a? *

Copy and paste relevant sections from the manuscript (include quotes in quotation marks "like this" to indicate direct quotes from your manuscript), or elaborate on this item by providing additional information not in the ms, or briefly explain why the item is not applicable/relevant for your study

The study was a randomised, controlled, non-inferiority trial comparing B-CBT to FtF-CBT

3b) Important changes to methods after trial commencement (such as eligibility criteria), with reasons

Does your paper address CONSORT subitem 3b? *

Copy and paste relevant sections from the manuscript (include quotes in quotation marks "like this" to indicate direct quotes from your manuscript), or elaborate on this item by providing additional information not in the ms, or briefly explain why the item is not applicable/relevant for your study

No important changes were made to methods after trial commencement

3b-i) Bug fixes, Downtimes, Content Changes

Bug fixes, Downtimes, Content Changes: ehealth systems are often dynamic systems. A description of changes to methods therefore also includes important changes made on the intervention or comparator during the trial (e.g., major bug fixes or changes in the functionality or content) (5-iii) and other “unexpected events” that may have influenced study design such as staff changes, system failures/downtimes, etc. [2].

	1	2	3	4	5	
subitem not at all important	○	○	○	◉	○	essential

Ryd markering

Does your paper address subitem 3b-i?

Copy and paste relevant sections from the manuscript (include quotes in quotation marks "like this" to indicate direct quotes from your manuscript), or elaborate on this item by providing additional information not in the ms, or briefly explain why the item is not applicable/relevant for your study

No important downtimes or system changes occurred after commencement of trial

4a) Eligibility criteria for participants

Does your paper address CONSORT subitem 4a? *

Copy and paste relevant sections from the manuscript (include quotes in quotation marks "like this" to indicate direct quotes from your manuscript), or elaborate on this item by providing additional information not in the ms, or briefly explain why the item is not applicable/relevant for your study

"All participants were eighteen years of age or older and met the diagnostic criteria for major depressive disorder according to the Diagnostic and Statistical Manual of Mental disorders 4th edition (DSM-IV) (34) as assessed by clinical psychologists. The diagnosis was confirmed by the research team using the semi-structured interview Mini International Neuropsychiatric Interview (35). Furthermore, a score of at least five on the Patient Health Questionnaire-9 (PHQ-9) (36) was required. Patients were excluded in case of current high risk of suicide or if they suffered from a co-morbid substance dependence, bipolar disorder, psychotic illness, or obsessive-compulsive disorder. Additionally, participants were excluded if they concurrently received psychological treatment for depression. They were also required to comprehend the Danish language and have access to a PC and Internet connection. Finally, they needed to be able and willing to travel to the physical location of the trial even if they were randomised to the face-to-face condition."

4a-i) Computer / Internet literacy

Computer / Internet literacy is often an implicit "de facto" eligibility criterion - this should be explicitly clarified.

	1	2	3	4	5	
subitem not at all important	○	○	○	◉	○	essential

Ryd markering

Does your paper address subitem 4a-i?

Copy and paste relevant sections from the manuscript (include quotes in quotation marks "like this" to indicate direct quotes from your manuscript), or elaborate on this item by providing additional information not in the ms, or briefly explain why the item is not applicable/relevant for your study

Dit svar

4a-ii) Open vs. closed, web-based vs. face-to-face assessments:

Open vs. closed, web-based vs. face-to-face assessments: Mention how participants were recruited (online vs. offline), e.g., from an open access website or from a clinic, and clarify if this was a purely web-based trial, or there were face-to-face components (as part of the intervention or for assessment), i.e., to what degree got the study team to know the participant. In online-only trials, clarify if participants were quasi-anonymous and whether having multiple identities was possible or whether technical or logistical measures (e.g., cookies, email confirmation, phone calls) were used to detect/prevent these.

	1	2	3	4	5	
subitem not at all important	○	○	○	○	○	essential

Does your paper address subitem 4a-ii? *

Copy and paste relevant sections from the manuscript (include quotes in quotation marks "like this" to indicate direct quotes from your manuscript), or elaborate on this item by providing additional information not in the ms, or briefly explain why the item is not applicable/relevant for your study

"They were also required to comprehend the Danish language and have access to a PC and Internet connection. "

4a-iii) Information giving during recruitment

Information given during recruitment. Specify how participants were briefed for recruitment and in the informed consent procedures (e.g., publish the informed consent documentation as appendix, see also item X26), as this information may have an effect on user self-selection, user expectation and may also bias results.

	1	2	3	4	5	
subitem not at all important	○	○	○	●	○	essential

Ryd markering

Does your paper address subitem 4a-iii?

Copy and paste relevant sections from the manuscript (include quotes in quotation marks "like this" to indicate direct quotes from your manuscript), or elaborate on this item by providing additional information not in the ms, or briefly explain why the item is not applicable/relevant for your study

"All participants received both written and oral information about the project and signed written informed consent prior to participation in the study."

4b) Settings and locations where the data were collected

Does your paper address CONSORT subitem 4b? *

Copy and paste relevant sections from the manuscript (include quotes in quotation marks "like this" to indicate direct quotes from your manuscript), or elaborate on this item by providing additional information not in the ms, or briefly explain why the item is not applicable/relevant for your study

"Data were stored by Odense Patient data Exploratory Network (OPEN) (43). Data were collected, transferred, and stored securely electronically as approved by the Danish Data Protection Agency (J.nr.: 14/26634 Reg. nr.: 2008-58-0035)."

4b-i) Report if outcomes were (self-)assessed through online questionnaires

Clearly report if outcomes were (self-)assessed through online questionnaires (as common in web-based trials) or otherwise.

	1	2	3	4	5	
subitem not at all important	○	○	○	◉	○	essential

Ryd markering

Does your paper address subitem 4b-i? *

Copy and paste relevant sections from the manuscript (include quotes in quotation marks "like this" to indicate direct quotes from your manuscript), or elaborate on this item by providing additional information not in the ms, or briefly explain why the item is not applicable/relevant for your study

"The Patient Health Questionnaire-9 (PHQ-9) (36) was used as primary outcome. The PHQ-9 is a nine items questionnaire, which was developed to measure depressive symptomatology in the primary health care sector. The nine items are each scored on a 0–3 point scale with a total score ranging from 0–27 with higher scores indicating more severe depression. The PHQ-9 has been shown to have good psychometric properties (44).

A number of additional measures were administered to assess different aspects of the participants symptomatology and experience during the treatments. The Quick Inventory of Depressive Symptomatology Self-Report (QIDS-16-SR) (45,46) was used in addition to the PHQ-9 because it is a promising questionnaire for assessing depressive symptoms especially in specialised mental health care and to conduct secondary analyses of primary latent construct of interest; depression. To measure the patients' satisfaction with the treatments, the Client Satisfaction Questionnaire (CSQ-8) (47,48) was used. The Credibility and Expectancy Questionnaire (CEQ) (49) was used to measure the patients' expectancy and judgement of credibility of the treatments. Finally, the level of therapeutic alliance was measured using the Working Alliance Inventory (WAI-SR) (50–52) and was rated by both the participants and the clinicians. For further description of the measures used, we refer to Mathiasen et al. (2016) (40)."

4b-ii) Report how institutional affiliations are displayed

Report how institutional affiliations are displayed to potential participants [on ehealth media], as affiliations with prestigious hospitals or universities may affect volunteer rates, use, and reactions with regards to an intervention.(Not a required item – describe only if this may bias results)

	1	2	3	4	5	
subitem not at all important	○	○	◉	○	○	essential

Ryd markering

Does your paper address subitem 4b-ii?

Copy and paste relevant sections from the manuscript (include quotes in quotation marks "like this" to indicate direct quotes from your manuscript), or elaborate on this item by providing additional information not in the ms, or briefly explain why the item is not applicable/relevant for your study

"Participants were recruited from 1 March 2016 to 1 April 2018 from the iCBT clinic ‘Internetpsykiatrien’, which is situated within secondary mental health care (Centre for Tele-psychiatry) at the Mental Health Services of the Region of Southern Denmark (37,38). Internetpsykiatrien offers guided iCBT treatment for anxiety and depression with self-referral. "

5) The interventions for each group with sufficient details to allow replication, including how and when they were actually administered

5-i) Mention names, credential, affiliations of the developers, sponsors, and owners

Mention names, credential, affiliations of the developers, sponsors, and owners [6] (if authors/evaluators are owners or developer of the software, this needs to be declared in a “Conflict of interest” section or mentioned elsewhere in the manuscript).

	1	2	3	4	5	
subitem not at all important	○	○	○	◉	○	essential

Ryd markering

Does your paper address subitem 5-i?

Copy and paste relevant sections from the manuscript (include quotes in quotation marks "like this" to indicate direct quotes from your manuscript), or elaborate on this item by providing additional information not in the ms, or briefly explain why the item is not applicable/relevant for your study

"In the blended condition six individual face-to-face CBT sessions were alternated with six to eight online CBT modules delivered through an Internet based treatment program. The program used was NoDep, which is based on CBT for depression including six mandatory modules and two optional ones. The core components consisted of psychoeducation, cognitive restructuring, restructuring of beliefs, behavioural activation and relapse prevention. Detailed descriptions have been provided elsewhere (39,40).

The same interventions were provided in the FtF-CBT group, but all delivered via face-to-face consultations. Both treatment conditions were described in one common treatment protocol thus ensuring similar treatment content and order of interventions across the two groups.

Licensed clinical psychologists or psychologists under supervision of the primary researcher (KM), who is also a licensed clinical psychologist, delivered all face-to-face consultations. To assess clinician adherence, (41) all face-to-face sessions were audio recorded and twenty sessions were randomly selected and evaluated by an external clinical expert (clinical psychologist and PhD with many years' experience). Clinician adherence was defined as the amount of prescribed interventions that were proscribed in the session rated on a five-point scale ranging from none (1) to all (5) (42).

To increase participant adherence, participants received automated reminders of homework assignments and questionnaires. Furthermore, in case a participant was inactive, he or she would be contacted by telephone or email. Additionally, in case a participant was unwilling or -able to engage with the program at home, a computer was set up at the clinic, for participants to engage with the online program on site. This was never used, however."

5-ii) Describe the history/development process

Describe the history/development process of the application and previous formative evaluations (e.g., focus groups, usability testing), as these will have an impact on adoption/use rates and help with interpreting results.

	1	2	3	4	5	
subitem not at all important	○	○	○	◉	○	essential

Ryd markering

Does your paper address subitem 5-ii?

Copy and paste relevant sections from the manuscript (include quotes in quotation marks "like this" to indicate direct quotes from your manuscript), or elaborate on this item by providing additional information not in the ms, or briefly explain why the item is not applicable/relevant for your study

"The program used was NoDep, which is based on CBT for depression including six mandatory modules and two optional ones. The core components consisted of psychoeducation, cognitive restructuring, restructuring of beliefs, behavioural activation and relapse prevention. Detailed descriptions have been provided elsewhere (39,40)"

5-iii) Revisions and updating

Revisions and updating. Clearly mention the date and/or version number of the application/intervention (and comparator, if applicable) evaluated, or describe whether the intervention underwent major changes during the evaluation process, or whether the development and/or content was “frozen” during the trial. Describe dynamic components such as news feeds or changing content which may have an impact on the replicability of the intervention (for unexpected events see item 3b).

	1	2	3	4	5	
subitem not at all important	○	○	○	◉	○	essential

Ryd markering

Does your paper address subitem 5-iii?

Copy and paste relevant sections from the manuscript (include quotes in quotation marks "like this" to indicate direct quotes from your manuscript), or elaborate on this item by providing additional information not in the ms, or briefly explain why the item is not applicable/relevant for your study

"No important changes were made to the program or the protocol during the trial."

5-iv) Quality assurance methods

Provide information on quality assurance methods to ensure accuracy and quality of information provided [1], if applicable.

	1	2	3	4	5	
subitem not at all important	○	○	○	◉	○	essential

Ryd markering

Does your paper address subitem 5-iv?

Copy and paste relevant sections from the manuscript (include quotes in quotation marks "like this" to indicate direct quotes from your manuscript), or elaborate on this item by providing additional information not in the ms, or briefly explain why the item is not applicable/relevant for your study

"Both treatment conditions were described in one common treatment protocol thus ensuring similar treatment content and order of interventions across the two groups.", "Licensed clinical psychologists or psychologists under supervision of the primary researcher (KM), who is also a licensed clinical psychologist, delivered all face-to-face consultations. To assess clinician adherence, (41) all face-to-face sessions were audio recorded and twenty sessions were randomly selected and evaluated by an external clinical expert (clinical psychologist and PhD with many years' experience). Clinician adherence was defined as the amount of prescribed interventions that were proscribed in the session rated on a five-point scale ranging from none (1) to all (5) (42)."

5-v) Ensure replicability by publishing the source code, and/or providing screenshots/screen-capture video, and/or providing flowcharts of the algorithms used

Ensure replicability by publishing the source code, and/or providing screenshots/screen-capture video, and/or providing flowcharts of the algorithms used. Replicability (i.e., other researchers should in principle be able to replicate the study) is a hallmark of scientific reporting.

	1	2	3	4	5	
subitem not at all important	○	○	○	◉	○	essential

Ryd markering

Does your paper address subitem 5-v?

Copy and paste relevant sections from the manuscript (include quotes in quotation marks "like this" to indicate direct quotes from your manuscript), or elaborate on this item by providing additional information not in the ms, or briefly explain why the item is not applicable/relevant for your study

The source code was proprietary and cannot be published by the research team.

5-vi) Digital preservation

Digital preservation: Provide the URL of the application, but as the intervention is likely to change or disappear over the course of the years; also make sure the intervention is archived (Internet Archive, webcitation.org, and/or publishing the source code or screenshots/videos alongside the article). As pages behind login screens cannot be archived, consider creating demo pages which are accessible without login.

	1	2	3	4	5	
subitem not at all important	○	○	○	◉	○	essential

Ryd markering

Does your paper address subitem 5-vi?

Copy and paste relevant sections from the manuscript (include quotes in quotation marks "like this" to indicate direct quotes from your manuscript), or elaborate on this item by providing additional information not in the ms, or briefly explain why the item is not applicable/relevant for your study

Since the program is proprietary, the access to the program is not open for all.

5-vii) Access

Access: Describe how participants accessed the application, in what setting/context, if they had to pay (or were paid) or not, whether they had to be a member of specific group. If known, describe how participants obtained “access to the platform and Internet” [1]. To ensure access for editors/reviewers/readers, consider to provide a “backdoor” login account or demo mode for reviewers/readers to explore the application (also important for archiving purposes, see vi).

	1	2	3	4	5	
subitem not at all important	○	○	○	○	○	essential

Does your paper address subitem 5-vii? *

Copy and paste relevant sections from the manuscript (include quotes in quotation marks "like this" to indicate direct quotes from your manuscript), or elaborate on this item by providing additional information not in the ms, or briefly explain why the item is not applicable/relevant for your study

"They were also required to comprehend the Danish language and have access to a PC and Internet connection. Finally, they needed to be able and willing to travel to the physical location of the trial even if they were randomised to the face-to-face condition.", "Access to the program was provided by the research team."

5-viii) Mode of delivery, features/functionalities/components of the intervention and comparator, and the theoretical framework

Describe mode of delivery, features/functionalities/components of the intervention and comparator, and the theoretical framework [6] used to design them (instructional strategy [1], behaviour change techniques, persuasive features, etc., see e.g., [7, 8] for terminology). This includes an in-depth description of the content (including where it is coming from and who developed it) [1]," whether [and how] it is tailored to individual circumstances and allows users to track their progress and receive feedback" [6]. This also includes a description of communication delivery channels and – if computer-mediated communication is a component – whether communication was synchronous or asynchronous [6]. It also includes information on presentation strategies [1], including page design principles, average amount of text on pages, presence of hyperlinks to other resources, etc. [1].

	1	2	3	4	5	
subitem not at all important	○	○	○	○	○	essential

Does your paper address subitem 5-viii? *

Copy and paste relevant sections from the manuscript (include quotes in quotation marks "like this" to indicate direct quotes from your manuscript), or elaborate on this item by providing additional information not in the ms, or briefly explain why the item is not applicable/relevant for your study

"Participants were recruited from 1 March 2016 to 1 April 2018 from the iCBT clinic 'Internetpsykiatrien', which is situated within secondary mental health care (Centre for Tele-psychiatry) at the Mental Health Services of the Region of Southern Denmark (37,38). ", "Access to the program was provided by the research team."

5-ix) Describe use parameters

Describe use parameters (e.g., intended "doses" and optimal timing for use). Clarify what instructions or recommendations were given to the user, e.g., regarding timing, frequency, heaviness of use, if any, or was the intervention used ad libitum.

	1	2	3	4	5	
subitem not at all important	○	○	○	◉	○	essential

Ryd markering

Does your paper address subitem 5-ix?

Copy and paste relevant sections from the manuscript (include quotes in quotation marks "like this" to indicate direct quotes from your manuscript), or elaborate on this item by providing additional information not in the ms, or briefly explain why the item is not applicable/relevant for your study

"In the blended condition six individual face-to-face CBT sessions were alternated with six to eight online CBT modules delivered through an Internet based treatment program. The program used was NoDep, which is based on CBT for depression including six mandatory modules and two optional ones. Both conditions were intended to last approximately twelve weeks. "

5-x) Clarify the level of human involvement

Clarify the level of human involvement (care providers or health professionals, also technical assistance) in the e-intervention or as co-intervention (detail number and expertise of professionals involved, if any, as well as “type of assistance offered, the timing and frequency of the support, how it is initiated, and the medium by which the assistance is delivered”. It may be necessary to distinguish between the level of human involvement required for the trial, and the level of human involvement required for a routine application outside of a RCT setting (discuss under item 21 – generalizability).

	1	2	3	4	5	
subitem not at all important	○	○	○	●	○	essential

Ryd markering

Does your paper address subitem 5-x?

Copy and paste relevant sections from the manuscript (include quotes in quotation marks "like this" to indicate direct quotes from your manuscript), or elaborate on this item by providing additional information not in the ms, or briefly explain why the item is not applicable/relevant for your study

"In the blended condition six individual face-to-face CBT sessions were alternated with six to eight online CBT modules delivered through an Internet based treatment program. The program used was NoDep, which is based on CBT for depression including six mandatory modules and two optional ones. Both conditions were intended to last approximately twelve weeks. "

5-xi) Report any prompts/reminders used

Report any prompts/reminders used: Clarify if there were prompts (letters, emails, phone calls, SMS) to use the application, what triggered them, frequency etc. It may be necessary to distinguish between the level of prompts/reminders required for the trial, and the level of prompts/reminders for a routine application outside of a RCT setting (discuss under item 21 – generalizability).

	1	2	3	4	5	
subitem not at all important	○	○	○	●	○	essential

Ryd markering

Does your paper address subitem 5-xi? *

Copy and paste relevant sections from the manuscript (include quotes in quotation marks "like this" to indicate direct quotes from your manuscript), or elaborate on this item by providing additional information not in the ms, or briefly explain why the item is not applicable/relevant for your study

"To increase participant adherence, participants received automated reminders of homework assignments and questionnaires. Furthermore, in case a participant was inactive, he or she would be contacted by telephone or email. Additionally, in case a participant was unwilling or -able to engage with the program at home, a computer was set up at the clinic, for participants to engage with the online program on site. This was never used, however. "

5-xii) Describe any co-interventions (incl. training/support)

Describe any co-interventions (incl. training/support): Clearly state any interventions that are provided in addition to the targeted eHealth intervention, as ehealth intervention may not be designed as stand-alone intervention. This includes training sessions and support [1]. It may be necessary to distinguish between the level of training required for the trial, and the level of training for a routine application outside of a RCT setting (discuss under item 21 – generalizability.

	1	2	3	4	5	
subitem not at all important	○	○	○	○	○	essential

Does your paper address subitem 5-xii? *

Copy and paste relevant sections from the manuscript (include quotes in quotation marks "like this" to indicate direct quotes from your manuscript), or elaborate on this item by providing additional information not in the ms, or briefly explain why the item is not applicable/relevant for your study

Since both conditions included face-to-face consultations, training per se was not needed.

6a) Completely defined pre-specified primary and secondary outcome measures, including how and when they were assessed

Does your paper address CONSORT subitem 6a? *

Copy and paste relevant sections from the manuscript (include quotes in quotation marks "like this" to indicate direct quotes from your manuscript), or elaborate on this item by providing additional information not in the ms, or briefly explain why the item is not applicable/relevant for your study

"After consent was granted, baseline measures were administered prior to randomisation. Follow-up measurements were conducted three, six and twelve months after baseline. Additionally, weekly measures were provided during treatment. The questionnaire packages were administered online using a secure web application for building and managing online surveys (RedCap), except the weekly monitoring of the B-CBT group, which was administered by the treatment programme.

Data were stored by Odense Patient data Exploratory Network (OPEN) (43). Data were collected, transferred, and stored securely electronically as approved by the Danish Data Protection Agency (J.nr.: 14/26634 Reg. nr.: 2008-58-0035).

The Patient Health Questionnaire-9 (PHQ-9) (36) was used as primary outcome. The PHQ-9 is a nine items questionnaire, which was developed to measure depressive symptomatology in the primary health care sector. The nine items are each scored on a 0–3 point scale with a total score ranging from 0–27 with higher scores indicating more severe depression. The PHQ-9 has been shown to have good psychometric properties (44).

A number of additional measures were administered to assess different aspects of the participants symptomatology and experience during the treatments. The Quick Inventory of Depressive Symptomatology Self-Report (QIDS-16-SR) (45,46) was used in addition to the PHQ-9 because it is a promising questionnaire for assessing depressive symptoms especially in specialised mental health care and to conduct secondary analyses of primary latent construct of interest; depression. To measure the patients' satisfaction with the treatments, the Client Satisfaction Questionnaire (CSQ-8) (47,48) was used. The Credibility and Expectancy Questionnaire (CEQ) (49) was used to measure the patients' expectancy and judgement of credibility of the treatments. Finally, the level of therapeutic alliance was measured using the Working Alliance Inventory (WAI-SR) (50–52) and was rated by both the participants and the clinicians. For further description of the measures used, we refer to Mathiasen et al. (2016) (40)."

6a-i) Online questionnaires: describe if they were validated for online use and apply CHERRIES items to describe how the questionnaires were designed/deployed

If outcomes were obtained through online questionnaires, describe if they were validated for online use and apply CHERRIES items to describe how the questionnaires were designed/deployed [9].

	1	2	3	4	5	
subitem not at all important	(•)	( )	( )	( )	( )	essential

Ryd markering

Does your paper address subitem 6a-i?

Copy and paste relevant sections from manuscript text

"After consent was granted, baseline measures were administered prior to randomisation. Follow-up measurements were conducted three, six and twelve months after baseline. Additionally, weekly measures were provided during treatment. The questionnaire packages were administered online using a secure web application for building and managing online surveys (RedCap), except the weekly monitoring of the B-CBT group, which was administered by the treatment programme.

Data were stored by Odense Patient data Exploratory Network (OPEN) (43). Data were collected, transferred, and stored securely electronically as approved by the Danish Data Protection Agency (J.nr.: 14/26634 Reg. nr.: 2008-58-0035).

The Patient Health Questionnaire-9 (PHQ-9) (36) was used as primary outcome. The PHQ-9 is a nine items questionnaire, which was developed to measure depressive symptomatology in the primary health care sector. The nine items are each scored on a 0–3 point scale with a total score ranging from 0–27 with higher scores indicating more severe depression. The PHQ-9 has been shown to have good psychometric properties (44).

A number of additional measures were administered to assess different aspects of the participants symptomatology and experience during the treatments. The Quick Inventory of Depressive Symptomatology Self-Report (QIDS-16-SR) (45,46) was used in addition to the PHQ-9 because it is a promising questionnaire for assessing depressive symptoms especially in specialised mental health care and to conduct secondary analyses of primary latent construct of interest; depression. To measure the patients' satisfaction with the treatments, the Client Satisfaction Questionnaire (CSQ-8) (47,48) was used. The Credibility and Expectancy Questionnaire (CEQ) (49) was used to measure the patients' expectancy and judgement of credibility of the treatments. Finally, the level of therapeutic alliance was measured using the Working Alliance Inventory (WAI-SR) (50–52) and was rated by both the participants and the clinicians. For further description of the measures used, we refer to Mathiasen et al. (2016) (40)."

6a-ii) Describe whether and how "use" (including intensity of use/dosage) was defined/measured/monitored

Describe whether and how "use" (including intensity of use/dosage) was defined/measured/monitored (logins, logfile analysis, etc.). Use/adoption metrics are important process outcomes that should be reported in any ehealth trial.

	1	2	3	4	5	
subitem not at all important	○	○	○	◉	○	essential

Ryd markering

Does your paper address subitem 6a-ii?

Copy and paste relevant sections from manuscript text

Dit svar

6a-iii) Describe whether, how, and when qualitative feedback from participants was obtained

Describe whether, how, and when qualitative feedback from participants was obtained (e.g., through emails, feedback forms, interviews, focus groups).

	1	2	3	4	5	
subitem not at all important	○	○	○	○	○	essential

Does your paper address subitem 6a-iii?

Copy and paste relevant sections from manuscript text

Dit svar

6b) Any changes to trial outcomes after the trial commenced, with reasons

Does your paper address CONSORT subitem 6b? *

Copy and paste relevant sections from the manuscript (include quotes in quotation marks "like this" to indicate direct quotes from your manuscript), or elaborate on this item by providing additional information not in the ms, or briefly explain why the item is not applicable/relevant for your study

"No important changes were made to the program or the protocol during the trial."

7a) How sample size was determined

NPT: When applicable, details of whether and how the clustering by care provides or centers was addressed

7a-i) Describe whether and how expected attrition was taken into account when calculating the sample size

Describe whether and how expected attrition was taken into account when calculating the sample size.

	1	2	3	4	5	
subitem not at all important	○	○	○	○	○	essential

Does your paper address subitem 7a-i?

Copy and paste relevant sections from manuscript title (include quotes in quotation marks "like this" to indicate direct quotes from your manuscript), or elaborate on this item by providing additional information not in the ms, or briefly explain why the item is not applicable/relevant for your study

Dit svar

7b) When applicable, explanation of any interim analyses and stopping guidelines

Does your paper address CONSORT subitem 7b? *

Copy and paste relevant sections from the manuscript (include quotes in quotation marks "like this" to indicate direct quotes from your manuscript), or elaborate on this item by providing additional information not in the ms, or briefly explain why the item is not applicable/relevant for your study

No interim analyses were conducted

8a) Method used to generate the random allocation sequence

NPT: When applicable, how care providers were allocated to each trial group

Does your paper address CONSORT subitem 8a? *

Copy and paste relevant sections from the manuscript (include quotes in quotation marks "like this" to indicate direct quotes from your manuscript), or elaborate on this item by providing additional information not in the ms, or briefly explain why the item is not applicable/relevant for your study

". A random number generator (Random Allocation Software) was applied with an allocation ratio of 1:1. Block randomisation was used with block sizes varying from eight to fourteen allocations per block. "

8b) Type of randomisation; details of any restriction (such as blocking and block size)

Does your paper address CONSORT subitem 8b? *

Copy and paste relevant sections from the manuscript (include quotes in quotation marks "like this" to indicate direct quotes from your manuscript), or elaborate on this item by providing additional information not in the ms, or briefly explain why the item is not applicable/relevant for your study

". A random number generator (Random Allocation Software) was applied with an allocation ratio of 1:1. Block randomisation was used with block sizes varying from eight to fourteen allocations per block. "

9) Mechanism used to implement the random allocation sequence (such as sequentially numbered containers), describing any steps taken to conceal the sequence until interventions were assigned

Does your paper address CONSORT subitem 9? *

Copy and paste relevant sections from the manuscript (include quotes in quotation marks "like this" to indicate direct quotes from your manuscript), or elaborate on this item by providing additional information not in the ms, or briefly explain why the item is not applicable/relevant for your study

"An independent researcher from the EU project E-Compared (33), who was not involved in the trial, performed the randomisation at an individual level, stratified by country after eligibility and baseline measurement. "

10) Who generated the random allocation sequence, who enrolled participants, and who assigned participants to interventions

Does your paper address CONSORT subitem 10? *

Copy and paste relevant sections from the manuscript (include quotes in quotation marks "like this" to indicate direct quotes from your manuscript), or elaborate on this item by providing additional information not in the ms, or briefly explain why the item is not applicable/relevant for your study

"An independent researcher from the EU project E-Compared (33), who was not involved in the trial, performed the randomisation at an individual level, stratified by country after eligibility and baseline measurement. "

11a) If done, who was blinded after assignment to interventions (for example, participants, care providers, those assessing outcomes) and how

NPT: Whether or not administering co-interventions were blinded to group assignment

11a-i) Specify who was blinded, and who wasn't

Specify who was blinded, and who wasn't. Usually, in web-based trials it is not possible to blind the participants [1, 3] (this should be clearly acknowledged), but it may be possible to blind outcome assessors, those doing data analysis or those administering co-interventions (if any).

	1	2	3	4	5	
subitem not at all important	○	○	○	◉	○	essential

Ryd markering

Does your paper address subitem 11a-i? *

Copy and paste relevant sections from the manuscript (include quotes in quotation marks "like this" to indicate direct quotes from your manuscript), or elaborate on this item by providing additional information not in the ms, or briefly explain why the item is not applicable/relevant for your study

"It was not possible to blind the patients nor the treating clinicians to the allocated treatment. However, those assessing the participants were blinded to allocation as was the researchers and statisticians involved up until the point of interpretation of results. Some questionnaires were only administered to the B-CBT group, and were kept in a separate dataset."

11a-ii) Discuss e.g., whether participants knew which intervention was the “intervention of interest” and which one was the “comparator”

Informed consent procedures (4a-ii) can create biases and certain expectations - discuss e.g., whether participants knew which intervention was the “intervention of interest” and which one was the “comparator”.

	1	2	3	4	5	
subitem not at all important	○	○	○	○	○	essential

Does your paper address subitem 11a-ii?

Copy and paste relevant sections from the manuscript (include quotes in quotation marks "like this" to indicate direct quotes from your manuscript), or elaborate on this item by providing additional information not in the ms, or briefly explain why the item is not applicable/relevant for your study

Dit svar

11b) If relevant, description of the similarity of interventions

(this item is usually not relevant for ehealth trials as it refers to similarity of a placebo or sham intervention to a active medication/intervention)

Does your paper address CONSORT subitem 11b? *

Copy and paste relevant sections from the manuscript (include quotes in quotation marks "like this" to indicate direct quotes from your manuscript), or elaborate on this item by providing additional information not in the ms, or briefly explain why the item is not applicable/relevant for your study

"In the blended condition six individual face-to-face CBT sessions were alternated with six to eight online CBT modules delivered through an Internet based treatment program. The program used was NoDep, which is based on CBT for depression including six mandatory modules and two optional ones. Both conditions were intended to last approximately twelve weeks. The core components consisted of psychoeducation, cognitive restructuring, restructuring of beliefs, behavioural activation and relapse prevention. Detailed descriptions have been provided elsewhere (39,40). No important changes were made to the program or the protocol during the trial."

12a) Statistical methods used to compare groups for primary and secondary outcomes

NPT: When applicable, details of whether and how the clustering by care providers or centers was addressed

Does your paper address CONSORT subitem 12a? *

Copy and paste relevant sections from the manuscript (include quotes in quotation marks "like this" to indicate direct quotes from your manuscript), or elaborate on this item by providing additional information not in the ms, or briefly explain why the item is not applicable/relevant for your study

"Primary analysis
For the primary analyses a linear multilevel mixed-effects model with restricted maximum likelihood estimator was used as intention-to-treat analyses. PHQ-9 scores were used as response variable. Time was included as a fixed effect and as a random effect nested within subject (random slope and intercept) (53). Correlation between slope and intercept was assumed. All available data were included.

All inferences assumed normally distributed error terms and heteroscedasticity, which were substantiated by visual inspection of a q-q normality plot and a plot of fitted values vs. standardised residuals.

Remission was defined as a score of <5 on the PHQ-9. Response to treatment was defined as >= 50% reduction on the PHQ-9.

The non-inferiority margin was set to d=.2.

Acceptability
Acceptability was estimated from measures of client satisfaction (CSQ-8) and working alliance as reported by the participants (WAI-SR, TAI) and the clinicians (WAIc). Means were compared using t-tests on raw scores using case-wise deletion in case of missing data.

Predictor Analyses
Mixed-effects models using all available data were applied for analyses of interactions between group and baseline variables by intention-to-treat principle. One model per predictor was used with PHQ-9 as the response variable in a series of univariate analyses. This was done to test whether baseline characteristics affected outcome differently in the two treatments. Inclusion of all parameters would have overfitted the model due to sample size. Time was included as both a fixed effect and as a random effect nested in individuals identically to the primary analysis.
Secondly, analyses of predictors of symptomatic change in the total sample were conducted also using a mixed effects model with PHQ-9 as response variable. Both multivariate and a series of univariate analyses were conducted. No group interaction was included in these analyses.

Completion
Having completed n >= 9 (75%) sessions was counted as completion and mean completion rates were compared between the groups by use of t-test. The completion rate of the B-CBT group included the sum of online modules and FtF sessions attended.

To assess the odds of non-completion predicted from the participants' baseline characteristics, a multivariate logistic regression analysis was conducted. As the response variable, a dichotomous variable for completion was used. Additionally, univariate logistic regression analyses were conducted using one model per predictor to investigate whether non-completion was predicted differently between the FtF-CBT treatment and the B-CBT which included an interaction term with group. Missing data were handled by multiply chained equation imputation.

All calculations were performed using R version 3.4.4 (54). Mixed-effects linear models were calculated using the lmerTest package (55), which fits models by use of the lme4 package (56) and provides P-values by use of Satterthwaite's degrees of freedom method. Two-way analyses were used with P<.05 as the threshold for significance for inferential statistics. All confidence intervals were calculated by bootstrapping using boot.ci (54).
"

12a-i) Imputation techniques to deal with attrition / missing values

Imputation techniques to deal with attrition / missing values: Not all participants will use the intervention/comparator as intended and attrition is typically high in ehealth trials. Specify how participants who did not use the application or dropped out from the trial were treated in the statistical analysis (a complete case analysis is strongly discouraged, and simple imputation techniques such as LOCF may also be problematic [4]).

	1	2	3	4	5	
subitem not at all important	○	○	○	◉	○	essential

Ryd markering

Does your paper address subitem 12a-i? *

Copy and paste relevant sections from the manuscript (include quotes in quotation marks "like this" to indicate direct quotes from your manuscript), or elaborate on this item by providing additional information not in the ms, or briefly explain why the item is not applicable/relevant for your study

"Missing values were handled by use of mixed-effects models including all available data."

12b) Methods for additional analyses, such as subgroup analyses and adjusted analyses

Does your paper address CONSORT subitem 12b? *

Copy and paste relevant sections from the manuscript (include quotes in quotation marks "like this" to indicate direct quotes from your manuscript), or elaborate on this item by providing additional information not in the ms, or briefly explain why the item is not applicable/relevant for your study

"Completion
Having completed n >= 9 (75%) sessions was counted as completion and mean completion rates were compared between the groups by use of t-test. The completion rate of the B-CBT group included the sum of online modules and FtF sessions attended.

To assess the odds of non-completion predicted from the participants' baseline characteristics, a multivariate logistic regression analysis was conducted. As the response variable, a dichotomous variable for completion was used. Additionally, univariate logistic regression analyses were conducted using one model per predictor to investigate whether non-completion was predicted differently between the FtF-CBT treatment and the B-CBT which included an interaction term with group. Missing data were handled by multiply chained equation imputation.

All calculations were performed using R version 3.4.4 (54). Mixed-effects linear models were calculated using the lmerTest package (55), which fits models by use of the lme4 package (56) and provides P-values by use of Satterthwaite's degrees of freedom method. Two-way analyses were used with P<.05 as the threshold for significance for inferential statistics. All confidence intervals were calculated by bootstrapping using boot.ci (54)."

X26) REB/IRB Approval and Ethical Considerations [recommended as subheading under "Methods"] (not a CONSORT item)

X26-i) Comment on ethics committee approval

	1	2	3	4	5	
subitem not at all important	○	○	○	○	○	essential

Does your paper address subitem X26-i?

Copy and paste relevant sections from the manuscript (include quotes in quotation marks "like this" to indicate direct quotes from your manuscript), or elaborate on this item by providing additional information not in the ms, or briefly explain why the item is not applicable/relevant for your study

Dit svar

x26-ii) Outline informed consent procedures

Outline informed consent procedures e.g., if consent was obtained offline or online (how? Checkbox, etc.?), and what information was provided (see 4a-ii). See [6] for some items to be included in informed consent documents.

	1	2	3	4	5	
subitem not at all important	○	○	○	○	○	essential

Does your paper address subitem X26-ii?

Copy and paste relevant sections from the manuscript (include quotes in quotation marks "like this" to indicate direct quotes from your manuscript), or elaborate on this item by providing additional information not in the ms, or briefly explain why the item is not applicable/relevant for your study

Dit svar

X26-iii) Safety and security procedures

Safety and security procedures, incl. privacy considerations, and any steps taken to reduce the likelihood or detection of harm (e.g., education and training, availability of a hotline)

	1	2	3	4	5	
subitem not at all important	○	○	○	○	○	essential

Does your paper address subitem X26-iii?

Copy and paste relevant sections from the manuscript (include quotes in quotation marks "like this" to indicate direct quotes from your manuscript), or elaborate on this item by providing additional information not in the ms, or briefly explain why the item is not applicable/relevant for your study

Dit svar

RESULTS

13a) For each group, the numbers of participants who were randomly assigned, received intended treatment, and were analysed for the primary outcome

NPT: The number of care providers or centers performing the intervention in each group and the number of patients treated by each care provider in each center

Does your paper address CONSORT subitem 13a? *

Copy and paste relevant sections from the manuscript (include quotes in quotation marks "like this" to indicate direct quotes from your manuscript), or elaborate on this item by providing additional information not in the ms, or briefly explain why the item is not applicable/relevant for your study

A table gives a complete overview of recruited participants (Table 1) and a CONSORT flow-diagram lists the flow of all participants at all measurement points (Figure 1).

13b) For each group, losses and exclusions after randomisation, together with reasons

Does your paper address CONSORT subitem 13b? (NOTE: Preferably, this is shown in a CONSORT flow diagram) *

Copy and paste relevant sections from the manuscript (include quotes in quotation marks "like this" to indicate direct quotes from your manuscript), or elaborate on this item by providing additional information not in the ms, or briefly explain why the item is not applicable/relevant for your study

A table gives a complete overview of recruited participants (Table 1) and a CONSORT flow-diagram lists the flow of all participants at all measurement points (Figure 1).

13b-i) Attrition diagram

Strongly recommended: An attrition diagram (e.g., proportion of participants still logging in or using the intervention/comparator in each group plotted over time, similar to a survival curve) or other figures or tables demonstrating usage/dose/engagement.

	1	2	3	4	5	
subitem not at all important	○	○	○	○	○	essential

Does your paper address subitem 13b-i?

Copy and paste relevant sections from the manuscript or cite the figure number if applicable (include quotes in quotation marks "like this" to indicate direct quotes from your manuscript), or elaborate on this item by providing additional information not in the ms, or briefly explain why the item is not applicable/relevant for your study

Dit svar

14a) Dates defining the periods of recruitment and follow-up

Does your paper address CONSORT subitem 14a? *

Copy and paste relevant sections from the manuscript (include quotes in quotation marks "like this" to indicate direct quotes from your manuscript), or elaborate on this item by providing additional information not in the ms, or briefly explain why the item is not applicable/relevant for your study

A table gives a complete overview of recruited participants (Table 1) and a CONSORT flow-diagram lists the flow of all participants at all measurement points (Figure 1).

14a-i) Indicate if critical "secular events" fell into the study period

Indicate if critical "secular events" fell into the study period, e.g., significant changes in Internet resources available or "changes in computer hardware or Internet delivery resources"

	1	2	3	4	5	
subitem not at all important	○	○	○	○	○	essential

Does your paper address subitem 14a-i?

Copy and paste relevant sections from the manuscript (include quotes in quotation marks "like this" to indicate direct quotes from your manuscript), or elaborate on this item by providing additional information not in the ms, or briefly explain why the item is not applicable/relevant for your study

Dit svar

14b) Why the trial ended or was stopped (early)

Does your paper address CONSORT subitem 14b? *

Copy and paste relevant sections from the manuscript (include quotes in quotation marks "like this" to indicate direct quotes from your manuscript), or elaborate on this item by providing additional information not in the ms, or briefly explain why the item is not applicable/relevant for your study

The trial was not stopped early

15) A table showing baseline demographic and clinical characteristics for each group

NPT: When applicable, a description of care providers (case volume, qualification, expertise, etc.) and centers (volume) in each group

Does your paper address CONSORT subitem 15? *

Copy and paste relevant sections from the manuscript (include quotes in quotation marks "like this" to indicate direct quotes from your manuscript), or elaborate on this item by providing additional information not in the ms, or briefly explain why the item is not applicable/relevant for your study

A table gives a complete overview of recruited participants (Table 1) and a CONSORT flow-diagram lists the flow of all participants at all measurement points (Figure 1).

15-i) Report demographics associated with digital divide issues

In ehealth trials it is particularly important to report demographics associated with digital divide issues, such as age, education, gender, social-economic status, computer/Internet/ehealth literacy of the participants, if known.

	1	2	3	4	5	
subitem not at all important	○	○	○	●	○	essential

Ryd markering

Does your paper address subitem 15-i? *

Copy and paste relevant sections from the manuscript (include quotes in quotation marks "like this" to indicate direct quotes from your manuscript), or elaborate on this item by providing additional information not in the ms, or briefly explain why the item is not applicable/relevant for your study

A table gives a complete overview of recruited participants (Table 1) and a CONSORT flow-diagram lists the flow of all participants at all measurement points (Figure 1).

16) For each group, number of participants (denominator) included in each analysis and whether the analysis was by original assigned groups

16-i) Report multiple “denominators” and provide definitions

Report multiple “denominators” and provide definitions: Report N’s (and effect sizes) “across a range of study participation [and use] thresholds” [1], e.g., N exposed, N consented, N used more than x times, N used more than y weeks, N participants “used” the intervention/comparator at specific pre-defined time points of interest (in absolute and relative numbers per group). Always clearly define “use” of the intervention.

	1	2	3	4	5	
subitem not at all important	○	○	○	●	○	essential

Ryd markering

Does your paper address subitem 16-i? *

Copy and paste relevant sections from the manuscript (include quotes in quotation marks "like this" to indicate direct quotes from your manuscript), or elaborate on this item by providing additional information not in the ms, or briefly explain why the item is not applicable/relevant for your study

A table gives a complete overview of recruited participants (Table 1) and a CONSORT flow-diagram lists the flow of all participants at all measurement points (Figure 1). "In both groups, large changes of the mean scores within groups were observed on the primary outcome measure (PHQ-9). Within the FtF-CBT group, the mean score decreased from 16.05 (SE = 0.63) at baseline to 4.67 (SE = 0.62) at twelve months follow-up. Likewise, in the B-CBT group, the mean score reduced from 14.42 (SE = 0.69) to 6.32 (SE = 0.95). In both groups the within-group changes in mean scores from baseline to twelve-months follow-up revealed large standardized effect-sizes (dFtF-CBT =-2.04, dB-CBT =-1.57) (57–59). ", "Between groups, a non-significant trend in effect size was noted favouring the FtF-CBT group at three-months follow-up (d = -.5, CI -1.62- 0.62) but not at six-months (d = .03, CI -1.43- 1.49), where the difference had all but disappeared amounting to just 0.17 points on the PHQ-9 and stayed well within the non-inferiority margin of d=.2. At twelve months follow-up, a non-significant difference could be observed slightly favouring FtF-CBT (p=-0.42, CI -1.49;0.65). However, at all measurement points, the confidence intervals were wide, stretching beyond the non-inferiority margin, rendering it impossible to infer generalisability of the results of non-inferiority. The differences were small and non-significant with almost no difference at six months follow-up. The same picture was seen on the secondary outcome of the QIDS." Additionally a table (Table 2) provides an overview of all observed means and standard errors at all major measurement points.

16-ii) Primary analysis should be intent-to-treat

Primary analysis should be intent-to-treat, secondary analyses could include comparing only “users”, with the appropriate caveats that this is no longer a randomized sample (see 18-i).

	1	2	3	4	5	
subitem not at all important	○	○	○	○	○	essential

Does your paper address subitem 16-ii?

Copy and paste relevant sections from the manuscript (include quotes in quotation marks "like this" to indicate direct quotes from your manuscript), or elaborate on this item by providing additional information not in the ms, or briefly explain why the item is not applicable/relevant for your study

Dit svar

17a) For each primary and secondary outcome, results for each group, and the estimated effect size and its precision (such as 95% confidence interval)

Does your paper address CONSORT subitem 17a? *

Copy and paste relevant sections from the manuscript (include quotes in quotation marks "like this" to indicate direct quotes from your manuscript), or elaborate on this item by providing additional information not in the ms, or briefly explain why the item is not applicable/relevant for your study

"The primary analyses using linear mixed-effects models with the PHQ-9 as outcome variable confirmed the within-group improvements in both groups being significant (FtF-CBT: ß = -0.03, SE = 0.00, P < .001; B-CBT: ß = -0.02, SE = 0.00, P < .001), which was also the case for the QIDS scores (FtF-CBT ß = -0.02, SE = 0.00, P < .001; B-CBT ß = -0.01, SE = 0.00, p<0.001). Note that the beta values are small, as they represent the change on the outcome measure per day. Between groups, a very small but significant interaction effect was observed on the PHQ-9 (ß = 0.01, SE = 0.00, P = .026), indicating a slight advantage of the FtF-CBT group. However, this was not the case on the QIDS (ß = 0.01, SE=0.00, P = .052), which was just above the significance level." Additionally a table (Table 3) provides an overview of estimates, standard errors and p-values of the primary analysis.

17a-i) Presentation of process outcomes such as metrics of use and intensity of use

In addition to primary/secondary (clinical) outcomes, the presentation of process outcomes such as metrics of use and intensity of use (dose, exposure) and their operational definitions is critical. This does not only refer to metrics of attrition (13-b) (often a binary variable), but also to more continuous exposure metrics such as “average session length”. These must be accompanied by a technical description how a metric like a “session” is defined (e.g., timeout after idle time) [1] (report under item 6a).

	1	2	3	4	5	
subitem not at all important	○	○	○	○	○	essential

Does your paper address subitem 17a-i?

Copy and paste relevant sections from the manuscript (include quotes in quotation marks "like this" to indicate direct quotes from your manuscript), or elaborate on this item by providing additional information not in the ms, or briefly explain why the item is not applicable/relevant for your study

Dit svar

17b) For binary outcomes, presentation of both absolute and relative effect sizes is recommended

Does your paper address CONSORT subitem 17b? *

Copy and paste relevant sections from the manuscript (include quotes in quotation marks "like this" to indicate direct quotes from your manuscript), or elaborate on this item by providing additional information not in the ms, or briefly explain why the item is not applicable/relevant for your study

"In total, n = 53 (69.7%) completed the treatment; n = 27 (71.1%) of the FtF-CBT group and n = 26 (68.4%) of the B-CBT group. Completers as well as non-completers showed a significant effect of time (completers ß = -0.03, p < .001; non-completers ß = -0.03, p <.001). In an analysis of the total sample including a binary interaction term for completion, no significant interaction was seen (ß = 0.00, P = .429), which indicated that there was no difference in effect between completers and non-completers.

In the FtF-CBT group, a mean of M = 9.8 sessions was completed. In the B-CBT group, M = 9.2 sessions was completed. The mean difference was not significant (t(74) = -0.70, P = .486).
"

18) Results of any other analyses performed, including subgroup analyses and adjusted analyses, distinguishing pre-specified from exploratory

Does your paper address CONSORT subitem 18? *

Copy and paste relevant sections from the manuscript (include quotes in quotation marks "like this" to indicate direct quotes from your manuscript), or elaborate on this item by providing additional information not in the ms, or briefly explain why the item is not applicable/relevant for your study

"There was no significant difference in client satisfaction between the groups (Mean diff. = -2.18, t(39.36) = 2.16, P = .146). Furthermore, no significant difference in working alliance was observed when reported by the participants (Mean diff. = 2.31, t(50.08) = 1.14, P = .26). However, the difference between the groups was larger and significant when rated by the treating clinicians (Mean diff. = 6.27, t(58.51) = 3.68, P < .001).

The response rates at twelve-months based on the PHQ-9 were 83.3% (n = 25) in the FtF-CBT group and 63.6% (n = 14) in the B-CBT group. The remission rates at twelve months were 60% (n = 18) for the FtF-CBT group and 50% (n = 11) for the B-CBT group. When inspecting all individual slopes of the primary model, we found no negative individual slopes indicating that no participants deteriorated.

Finally, twenty randomly selected audio recorded sessions were examined for treatment fidelity by an external expert in clinical psychology. Among the sample, session numbers ranged from three to twelve, three out of four therapists were represented, and both groups were well represented with fourteen sessions being from the FtF-CBT group. The mean score of treatment fidelity was 4.25 (SD = 0.71) on a scale ranging for one to five.
", "In a multivariate analysis of the total sample, only being on sick leave and preferring blended care predicted outcome. Being on sick leave added to the slope (3.96; SE=1.54; p=0.015) i.e. produced a smaller reduction in symptom change. Preferring blended care subtracted from the slope estimate (-3.25; SE=1.53; p=0.041) thus signifying an increase in symptom reduction. However, no significant interaction effect with groups was observed. Table 4 summarises all predictor variables with standard errors and p-values from a multivariate analysis of the total sample.

In a series of univariate interaction analyses of each parameter times group we found significant interaction effect of being part-time employed (ß=-5.83, SE=2.68, p=0.034) or unemployed (ß=-7.59, SE=2.52, p=0.004) both favouring B-CBT." A table (Table 4) provides an overvies of prediction analysis.

In total, n = 53 (69.7%) completed the treatment; n = 27 (71.1%) of the FtF-CBT group and n = 26 (68.4%) of the B-CBT group. Completers as well as non-completers showed a significant effect of time (completers ß = -0.03, P < .001; non-completers ß = -0.03, P <.001). In an analysis of the total sample including a binary interaction term for completion, no significant interaction was seen (ß = 0.00, P = .429), which indicated that there was no difference in effect between completers and non-completers.

"In the FtF-CBT group, a mean of M = 9.8 sessions was completed. In the B-CBT group, M = 9.2 sessions was completed. The mean difference was not significant (t(74) = -0.70, P = .486)

We did not find that any variables significantly predicted non-completion in multivariate analyses of the total sample, nor did we find any interaction effect between any of the baseline characteristics and groups in a series of univariate analyses indicating no difference in risk of non-completion on any baseline characteristic between groups. "

18-i) Subgroup analysis of comparing only users

A subgroup analysis of comparing only users is not uncommon in ehealth trials, but if done, it must be stressed that this is a self-selected sample and no longer an unbiased sample from a randomized trial (see 16-iii).

	1	2	3	4	5	
subitem not at all important	○	○	○	○	○	essential

Does your paper address subitem 18-i?

Copy and paste relevant sections from the manuscript (include quotes in quotation marks "like this" to indicate direct quotes from your manuscript), or elaborate on this item by providing additional information not in the ms, or briefly explain why the item is not applicable/relevant for your study

Dit svar

19) All important harms or unintended effects in each group

(for specific guidance see CONSORT for harms)

Does your paper address CONSORT subitem 19? *

Copy and paste relevant sections from the manuscript (include quotes in quotation marks "like this" to indicate direct quotes from your manuscript), or elaborate on this item by providing additional information not in the ms, or briefly explain why the item is not applicable/relevant for your study

"When inspecting all individual slopes of the primary model, we found no negative individual slopes indicating that no participants deteriorated. "

19-i) Include privacy breaches, technical problems

Include privacy breaches, technical problems. This does not only include physical "harm" to participants, but also incidents such as perceived or real privacy breaches [1], technical problems, and other unexpected/unintended incidents. "Unintended effects" also includes unintended positive effects [2].

	1	2	3	4	5	
subitem not at all important	○	○	○	○	○	essential

Does your paper address subitem 19-i?

Copy and paste relevant sections from the manuscript (include quotes in quotation marks "like this" to indicate direct quotes from your manuscript), or elaborate on this item by providing additional information not in the ms, or briefly explain why the item is not applicable/relevant for your study

Dit svar

19-ii) Include qualitative feedback from participants or observations from staff/researchers

Include qualitative feedback from participants or observations from staff/researchers, if available, on strengths and shortcomings of the application, especially if they point to unintended/unexpected effects or uses. This includes (if available) reasons for why people did or did not use the application as intended by the developers.

	1	2	3	4	5	
subitem not at all important	○	○	○	○	○	essential

Does your paper address subitem 19-ii?

Copy and paste relevant sections from the manuscript (include quotes in quotation marks "like this" to indicate direct quotes from your manuscript), or elaborate on this item by providing additional information not in the ms, or briefly explain why the item is not applicable/relevant for your study

Dit svar

DISCUSSION

22) Interpretation consistent with results, balancing benefits and harms, and considering other relevant evidence

NPT: In addition, take into account the choice of the comparator, lack of or partial blinding, and unequal expertise of care providers or centers in each group

22-i) Restate study questions and summarize the answers suggested by the data, starting with primary outcomes and process outcomes (use)

Restate study questions and summarize the answers suggested by the data, starting with primary outcomes and process outcomes (use).

	1	2	3	4	5	
subitem not at all important	○	○	○	◉	○	essential

Ryd markering

Does your paper address subitem 22-i? *

Copy and paste relevant sections from the manuscript (include quotes in quotation marks "like this" to indicate direct quotes from your manuscript), or elaborate on this item by providing additional information not in the ms, or briefly explain why the item is not applicable/relevant for your study

"The main aim of the present study was to compare the clinical effectiveness of blended CBT to traditional FtF-CBT, since the blended format may hold the promise to combine advantages of the traditional and the new format of delivery. In the present study, we found very similar trajectories of improvement in both groups as well as on measures of other parameters such as working alliance and retention. However, it was possible to detect a significant difference between groups in slight favour of FTF-CBT."

22-ii) Highlight unanswered new questions, suggest future research

Highlight unanswered new questions, suggest future research.

	1	2	3	4	5	
subitem not at all important	○	○	○	○	○	essential

Does your paper address subitem 22-ii?

Copy and paste relevant sections from the manuscript (include quotes in quotation marks "like this" to indicate direct quotes from your manuscript), or elaborate on this item by providing additional information not in the ms, or briefly explain why the item is not applicable/relevant for your study

Dit svar

20) Trial limitations, addressing sources of potential bias, imprecision, and, if relevant, multiplicity of analyses

20-i) Typical limitations in ehealth trials

Typical limitations in ehealth trials: Participants in ehealth trials are rarely blinded. Ehealth trials often look at a multiplicity of outcomes, increasing risk for a Type I error. Discuss biases due to non-use of the intervention/usability issues, biases through informed consent procedures, unexpected events.

	1	2	3	4	5	
subitem not at all important	○	○	○	○	◉	essential

Ryd markering

Does your paper address subitem 20-i? *

Copy and paste relevant sections from the manuscript (include quotes in quotation marks "like this" to indicate direct quotes from your manuscript), or elaborate on this item by providing additional information not in the ms, or briefly explain why the item is not applicable/relevant for your study

"The present study compared directly the formats of delivery with a minimum of the variance explained by differences in therapeutic methods, which is both a strength and a weakness of the design. While it lends itself well to compare the two treatment formats, it also somewhat limits the ecological validity making it more difficult to make inferences about the clinical effect in routine care. Furthermore, since the study recruited from Internetpsykiatrien which offers self-referral even though the clinic is situated in secondary care, it can be difficult to generalise to future implementations. Finally, due to the small sample size the study has difficulty inferring non-inferiority even if the many observations and advanced statistical procedures appear to have compensated for that to some degree. The large EU-study E-compared will be able to pool data from many studies, including the present one, and may thus be able to reach more robust conclusions about non-inferiority. "

21) Generalisability (external validity, applicability) of the trial findings

NPT: External validity of the trial findings according to the intervention, comparators, patients, and care providers or centers involved in the trial

21-i) Generalizability to other populations

Generalizability to other populations: In particular, discuss generalizability to a general Internet population, outside of a RCT setting, and general patient population, including applicability of the study results for other organizations

	1	2	3	4	5	
subitem not at all important	○	○	○	○	○	essential

Does your paper address subitem 21-i?

Copy and paste relevant sections from the manuscript (include quotes in quotation marks "like this" to indicate direct quotes from your manuscript), or elaborate on this item by providing additional information not in the ms, or briefly explain why the item is not applicable/relevant for your study

Dit svar

21-ii) Discuss if there were elements in the RCT that would be different in a routine application setting

Discuss if there were elements in the RCT that would be different in a routine application setting (e.g., prompts/reminders, more human involvement, training sessions or other co-interventions) and what impact the omission of these elements could have on use, adoption, or outcomes if the intervention is applied outside of a RCT setting.

	1	2	3	4	5	
subitem not at all important	○	○	○	○	○	essential

Does your paper address subitem 21-ii?

Copy and paste relevant sections from the manuscript (include quotes in quotation marks "like this" to indicate direct quotes from your manuscript), or elaborate on this item by providing additional information not in the ms, or briefly explain why the item is not applicable/relevant for your study

Dit svar

OTHER INFORMATION

23) Registration number and name of trial registry

Does your paper address CONSORT subitem 23? *

Copy and paste relevant sections from the manuscript (include quotes in quotation marks "like this" to indicate direct quotes from your manuscript), or elaborate on this item by providing additional information not in the ms, or briefly explain why the item is not applicable/relevant for your study

"The trial was registered with ClinicalTrials.gov NCT02796573."

24) Where the full trial protocol can be accessed, if available

Does your paper address CONSORT subitem 24? *

Cite a Multimedia Appendix, other reference, or copy and paste relevant sections from the manuscript (include quotes in quotation marks "like this" to indicate direct quotes from your manuscript), or elaborate on this item by providing additional information not in the ms, or briefly explain why the item is not applicable/relevant for your study

"The trial protocol was published in BMC (40)."

25) Sources of funding and other support (such as supply of drugs), role of funders

Does your paper address CONSORT subitem 25? *

Copy and paste relevant sections from the manuscript (include quotes in quotation marks "like this" to indicate direct quotes from your manuscript), or elaborate on this item by providing additional information not in the ms, or briefly explain why the item is not applicable/relevant for your study

"Funding has been granted from the Research Fund of the Mental Health Services of Southern Denmark, J.B. Winsløvs Vej 20 indg. 220B, 5000 Odense C.
And from the Innovation Fund Denmark, Østergade 26 A, 4. sal, DK – 1100 København K as part of the project ENTER (ID nr.: 5159-00002B).
Both are public funds. None of the funds have had any role in the design of the study nor in collection of data, analysis of data, interpretation of data, or writing the manuscript."

X27) Conflicts of Interest (not a CONSORT item)

X27-i) State the relation of the study team towards the system being evaluated

In addition to the usual declaration of interests (financial or otherwise), also state the relation of the study team towards the system being evaluated, i.e., state if the authors/evaluators are distinct from or identical with the developers/sponsors of the intervention.

	1	2	3	4	5	
subitem not at all important	○	○	○	○	○	essential

Does your paper address subitem X27-i?

Copy and paste relevant sections from the manuscript (include quotes in quotation marks "like this" to indicate direct quotes from your manuscript), or elaborate on this item by providing additional information not in the ms, or briefly explain why the item is not applicable/relevant for your study

Dit svar

About the CONSORT EHEALTH checklist

As a result of using this checklist, did you make changes in your manuscript? *

- ○ yes, major changes
- ◉ yes, minor changes
- ○ no

What were the most important changes you made as a result of using this checklist?

Dit svar

How much time did you spend on going through the checklist INCLUDING making changes in your manuscript *

I spend approximately 3 hours

As a result of using this checklist, do you think your manuscript has improved? *

- ◉ yes
- ○ no
- ○ Andet:

Would you like to become involved in the CONSORT EHEALTH group?

This would involve for example becoming involved in participating in a workshop and writing an "Explanation and Elaboration" document

- yes
- no
- Andet:

Any other comments or questions on CONSORT EHEALTH

Dit svar

STOP - Save this form as PDF before you click submit

To generate a record that you filled in this form, we recommend to generate a PDF of this page (on a Mac, simply select "print" and then select "print as PDF") before you submit it.

When you submit your (revised) paper to JMIR, please upload the PDF as supplementary file.

Don't worry if some text in the textboxes is cut off, as we still have the complete information in our database. Thank you!

Final step: Click submit !

Click submit so we have your answers in our database!

Send Ryd formular

Indsend aldrig adgangskoder via Google Analyse.

Dette indhold er hverken oprettet eller godkendt af Google. Rapportér misbrug - Servicevilkår - Privatlivspolitik

Google Analyse
